# Triptolide and its prodrug Minnelide target high-risk *MYC*-amplified medulloblastoma in preclinical models

**DOI:** 10.1172/JCI171136

**Published:** 2024-06-17

**Authors:** Jezabel Rodriguez-Blanco, April D. Salvador, Robert K. Suter, Marzena Swiderska-Syn, Isabel Palomo-Caturla, Valentin Kliebe, Pritika Shahani, Kendell Peterson, Maria Turos-Cabal, Megan E. Vieira, Daniel T. Wynn, Ashley J. Howell, Fan Yang, Yuguang Ban, Heather J. McCrea, Frederique Zindy, Etienne Danis, Rajeev Vibhakar, Anna Jermakowicz, Vanesa Martin, Christopher C. Coss, Brent T. Harris, Aguirre de Cubas, X. Steven Chen, Thibaut Barnoud, Martine F. Roussel, Nagi G. Ayad, David J. Robbins

**Affiliations:** 1 Darby Children’s Research Institute, Department of Pediatrics, and; 2 Hollings Cancer Center, Medical University of South Carolina, Charleston, South Carolina, USA.; 3 Department of Oncology, Lombardi Comprehensive Cancer Center, Georgetown University, Washington DC, USA.; 4 Department of Public Health Sciences, and; 5 Sylvester Comprehensive Cancer Center, University of Miami, Miller School of Medicine, Miami, Florida, USA.; 6 Departments of Neurological Surgery and Pediatrics, University of Miami, Jackson Health System, Miller School of Medicine, Miami, Florida, USA.; 7 Department of Tumor Cell Biology, St. Jude Children’s Research Hospital, Memphis, Tennessee, USA.; 8 University of Colorado Cancer Center,; 9 Department of Biomedical Informatics, and; 10 Department of Pediatrics, University of Colorado Anschutz Medical Campus, Aurora, Colorado, USA.; 11 Department of Morphology and Cell Biology, University of Oviedo, Oviedo, Asturias, Spain.; 12 College of Pharmacy, The Ohio State University, Columbus, Ohio, USA.; 13 Departments of Neurology and Pathology, Georgetown University Medical Center, Washington DC, USA.; 14 Department of Microbiology and Immunology, and; 15 Department of Biochemistry and Molecular Biology, Medical University of South Carolina, Charleston, South Carolina, USA.

**Keywords:** Oncology, Brain cancer

## Abstract

Most children with medulloblastoma (MB) achieve remission, but some face very aggressive metastatic tumors. Their dismal outcome highlights the critical need to advance therapeutic approaches that benefit such high-risk patients. Minnelide, a clinically relevant analog of the natural product triptolide, has oncostatic activity in both preclinical and early clinical settings. Despite its efficacy and tolerable toxicity, this compound has not been evaluated in MB. Utilizing a bioinformatic data set that integrates cellular drug response data with gene expression, we predicted that Group 3 (G3) MB, which has a poor 5-year survival, would be sensitive to triptolide/Minnelide. We subsequently showed that both triptolide and Minnelide attenuate the viability of G3 MB cells ex vivo. Transcriptomic analyses identified MYC signaling, a pathologically relevant driver of G3 MB, as a downstream target of this class of drugs. We validated this MYC dependency in G3 MB cells and showed that triptolide exerts its efficacy by reducing both *MYC* transcription and MYC protein stability. Importantly, Minnelide acted on MYC to reduce tumor growth and leptomeningeal spread, which resulted in improved survival of G3 MB animal models. Moreover, Minnelide improved the efficacy of adjuvant chemotherapy, further highlighting its potential for the treatment of MYC-driven G3 MB.

## Abstract 2

Minnelide, a prodrug of triptolide, targets MYC to attenuate the proliferation and leptomeningeal spread of group 3 medulloblastoma, supporting its translation for treatment of patients in this cohort.

## Graphical abstract

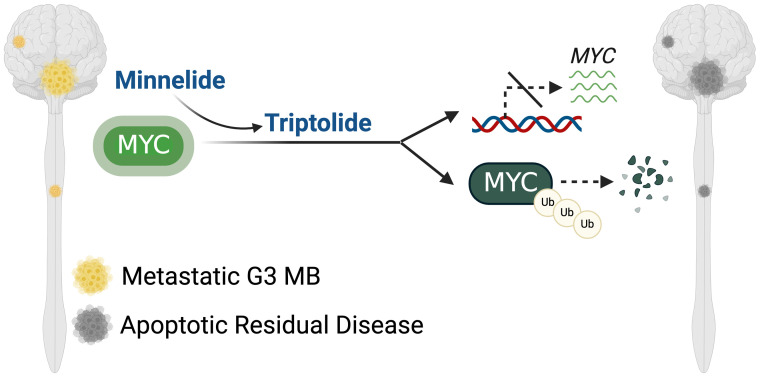

## Introduction

Nervous system tumors are the leading cause of cancer-related death in children (1), with medulloblastoma (MB) being the most common malignant form (2). Over the last decade, genomic stratification has identified 4 major molecular subgroups of MB: Wingless (WNT), Sonic Hedgehog (SHH), Group 3 (G3), and Group 4 (G4) (3). This subgrouping provides a template with which to begin identifying and testing subgroup-specific therapeutics. With an approximately 40% 5-year survival rate, patients with G3 MB harboring amplifications in *MYC* (formerly *C-MYC*) exhibit the worst prognosis among all MB subgroups (4). These patients often present with metastasis at the time of diagnosis and to some extent are resistant to standard-of-care treatments (4–6). Thus, patients with G3 MB have the greatest need for effective therapeutic approaches. Additionally, *MYC* amplifications are not only conserved at relapse, but also commonly emerge de novo in recurrent MB, either alone or in combination with *TP53* defects (7, 8). This observation is considered a characteristic of rapidly progressing disease (7), further emphasizing the urgent need for effective strategies to target MYC-driven MB.

Extracts from the vine *Tripterygium* *wilfordii* have been used in traditional Chinese medicine for the treatment of autoimmune and inflammatory diseases (9). Importantly, in preclinical studies, a diterpene triperoxide isolated from this vine, triptolide, was also shown to have potent antiproliferative effects in several types of tumors (10, 11). The efficacy of triptolide against one of the tumors with the poorest outcome, pancreatic ductal adenocarcinoma (10, 12), raised interest in the development of analogs with better medicinal chemical properties. One of the triptolide derivatives with improved solubility is the prodrug Minnelide, which is cleaved by alkaline phosphatases present in blood and other body tissues to release bioactive triptolide (11, 12). Minnelide was subsequently evaluated in phase I (ClinicalTrials.gov NCT01927965, NCT03347994, NCT05557851, NCT05566834, NCT01927965, NCT03760523, NCT03129139, and NCT05166616) and II (NCT03117920, NCT04896073) trials, where it showed tolerable toxicity as well as its potential to attenuate pancreatic tumor growth (13). Although the mechanisms by which triptolide and its analogs arrest tumor growth are not fully understood, MYC levels seem to predict sensitivity to triptolide, with those tumors harboring *MYC* amplifications showing the greatest sensitivity (14).

Due to the limited clinical options available to patients with MB, especially those with high-risk metastatic disease (15), a bioinformatic mining strategy was used to identify a subset of patients with MB that are likely to respond to triptolide. Consistent with previous response predictions in other tumors (14), these analyses highlighted G3 MB as the subgroup most likely to be triptolide/Minnelide sensitive. We subsequently validated this finding to show that G3 MB cell lines are approximately 100 times more sensitive to triptolide than SHH MB cells. This sensitivity of G3 MB cells to triptolide correlated with a decrease in *MYC* transcription and a reduction in the overall stability of this protein. In vivo, triptolide and Minnelide attenuated tumor growth and leptomeningeal spread, and increased symptom-free survival in mouse- and patient-derived G3 MB models. Further supporting its translational potential, Minnelide increased the in vivo efficacy of cyclophosphamide, a compound currently included in MB treatment protocols (16). Our work demonstrates that the clinically relevant triptolide derivative Minnelide reduces MYC-driven G3 MB progression and supports its clinical evaluation in the treatment of patients with high-risk metastatic G3 MB.

## Results

### Bioinformatic analysis predicts G3 MB response to triptolide.

**Figure 1:**
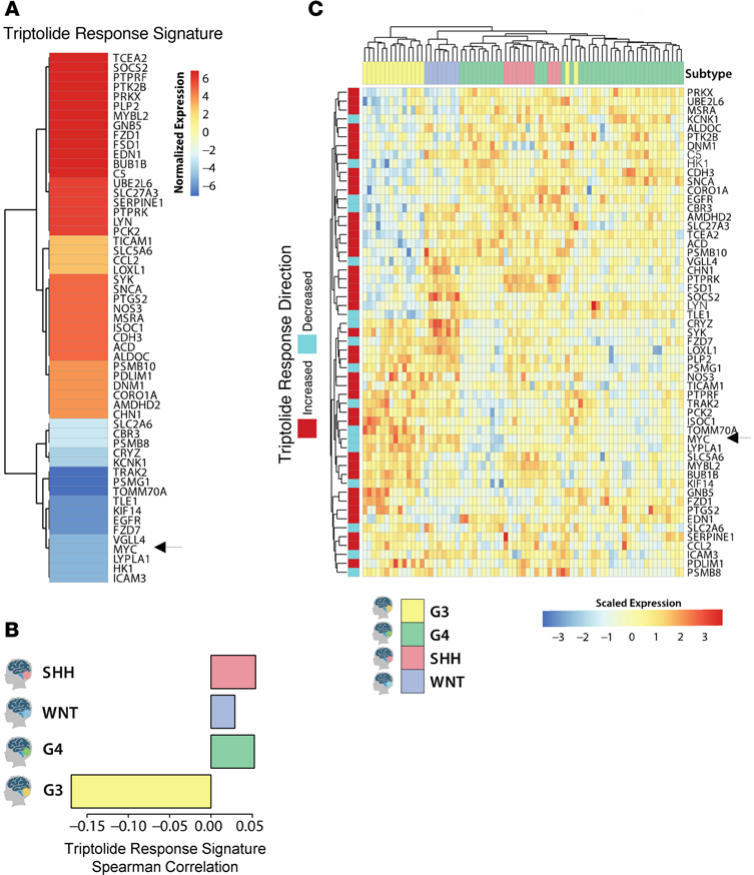
Bioinformatic analysis predicts that G3 MB will respond to triptolide. (**A**) Normalized expression of the 55 genes included within the NIH LINCS L1000 transcriptional consensus response signature for triptolide that overlap with MB subgroup–differentiating signatures is shown. The arrow highlights *MYC* within the downregulated genes in this signature. (**B**) Spearman’s correlation between the triptolide response gene signature was calculated against SHH, WNT, G3, and G4 disease signatures obtained through analysis of transcriptomic data included in the Robinson et al. 2012 data set. (**C**) Heatmap representing the expression of the top 55 triptolide response genes per MB subgroup. The arrow highlights *MYC* expression among MB subgroups.

To evaluate the efficacy of triptolide in targeting MB, we utilized an established bioinformatic portal, the NIH Library of Integrative Network–based Cellular Signatures (LINCS) L1000 data repository. This repository contains gene expression profiles for more than 1,000 cell lines treated with over 40,000 compounds (17). We mined this data set to identify a transcriptional consensus response signature to triptolide in cells (Figure 1A). We subsequently correlated this triptolide response signature with that of transcriptional signatures of each MB subgroup, to determine which subgroups might be responsive to triptolide compounds. While the triptolide signature showed positive correlations with the signatures of WNT, SHH, and G4 MB, it was negatively correlated with that of G3 MB (Figure 1B). This negative correlation suggests that triptolide disrupts the gene expression characterizing this subset of tumors and therefore predicts its response to triptolide. Additionally, hierarchical clustering of MB patient gene expression data using this triptolide response signature stratified G3 MB patients from those of the other subgroups (Figure 1C). These results suggest that G3 MB tumors could respond to triptolide.

**Figure 2:**
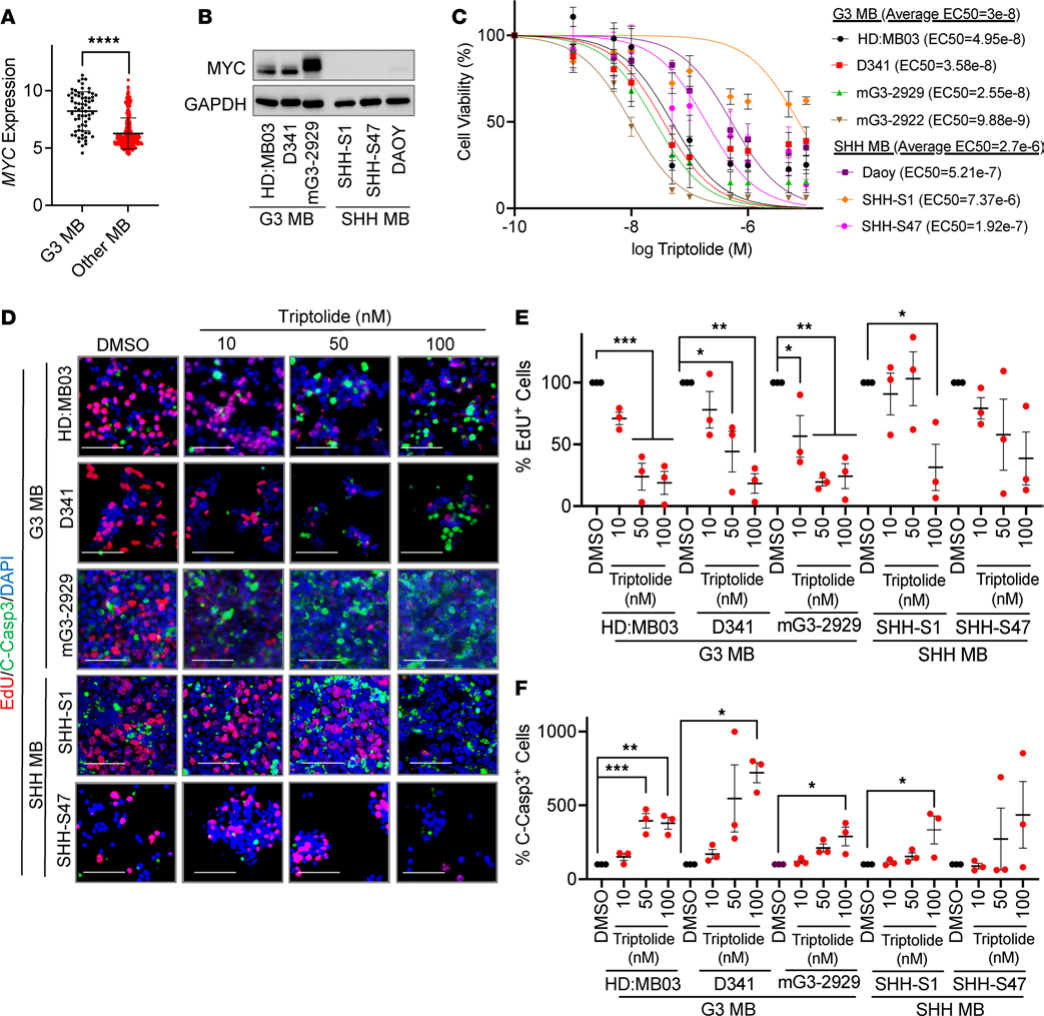
G3 MB cultures have an enhanced response to triptolide. (**A**) The Cavalli et al. 2017 data set was used to compare the expression of *MYC* in G3 MB patients versus the other MB subgroups. Expression data were analyzed using an unpaired, 1-tailed Student’s *t* test. (**B**) MYC levels were assessed by immunoblotting in 3 G3 and 3 SHH MB cultures. (**C**) G3 and SHH MB cells were incubated with increasing concentrations of triptolide for 48 hours before assaying cell viability using an MTT reduction assay. EC_50_ values were calculated using nonlinear regression analyses (G3 MB *n* = 4, SHH MB *n* = 3). (**D**) G3 and SHH MB cultures were exposed to the indicated concentrations of triptolide for 16 hours. Cell proliferation and apoptosis were assayed by EdU incorporation and cleaved Casp3 (C-Casp3) staining, respectively. Representative images (scale bars: 50 μm) are shown. (**E**) Quantification of the number of EdU-positive cells per field in similarly treated cultures (*n* = 3). (**F**) The number of C-Casp3–positive cells per field in similarly treated cultures was quantified (*n* = 3). Data presented as mean ± SEM. Data in **E** and **F** were normalized to DMSO and analyzed using 1-way ANOVA followed by Dunnett’s post hoc test. *P < 0.05; **P < 0.01; ***P < 0.001; ****P < 0.0001.

As *MYC* expression has been shown to correlate tumor response to triptolide treatment (14), we asked whether our L1000 data set–based predictions would align with this observation. Analysis of transcriptomic data (4) revealed higher *MYC* expression in tissues from patients classified as G3 MB (Figure 2A), where the expression of this oncogene also serves as an outcome predictor (Supplemental Figure 1A; supplemental material available online with this article; https://doi.org/10.1172/JCI171136DS1). We then treated G3 MB cells with increasing concentrations of triptolide using SHH MB cells, which show low MYC levels (Figure 2B), as a negative control. Cell viability analyses showed that triptolide attenuates the growth of G3 MB cultures, with a half maximal effective concentration (EC_50_) approximately 100 times lower than for SHH subgroup cells (Figure 2C). Furthermore, concentrations of triptolide capable of attenuating cell proliferation and triggering apoptosis in G3 MB cells had minimal effects on the propagation of SHH subgroup cells (Figure 2, D–F, and Supplemental Figure 1, B–F).

### Triptolide acts on MYC signaling to attenuate the growth of G3 MB cultures.

**Figure 3:**
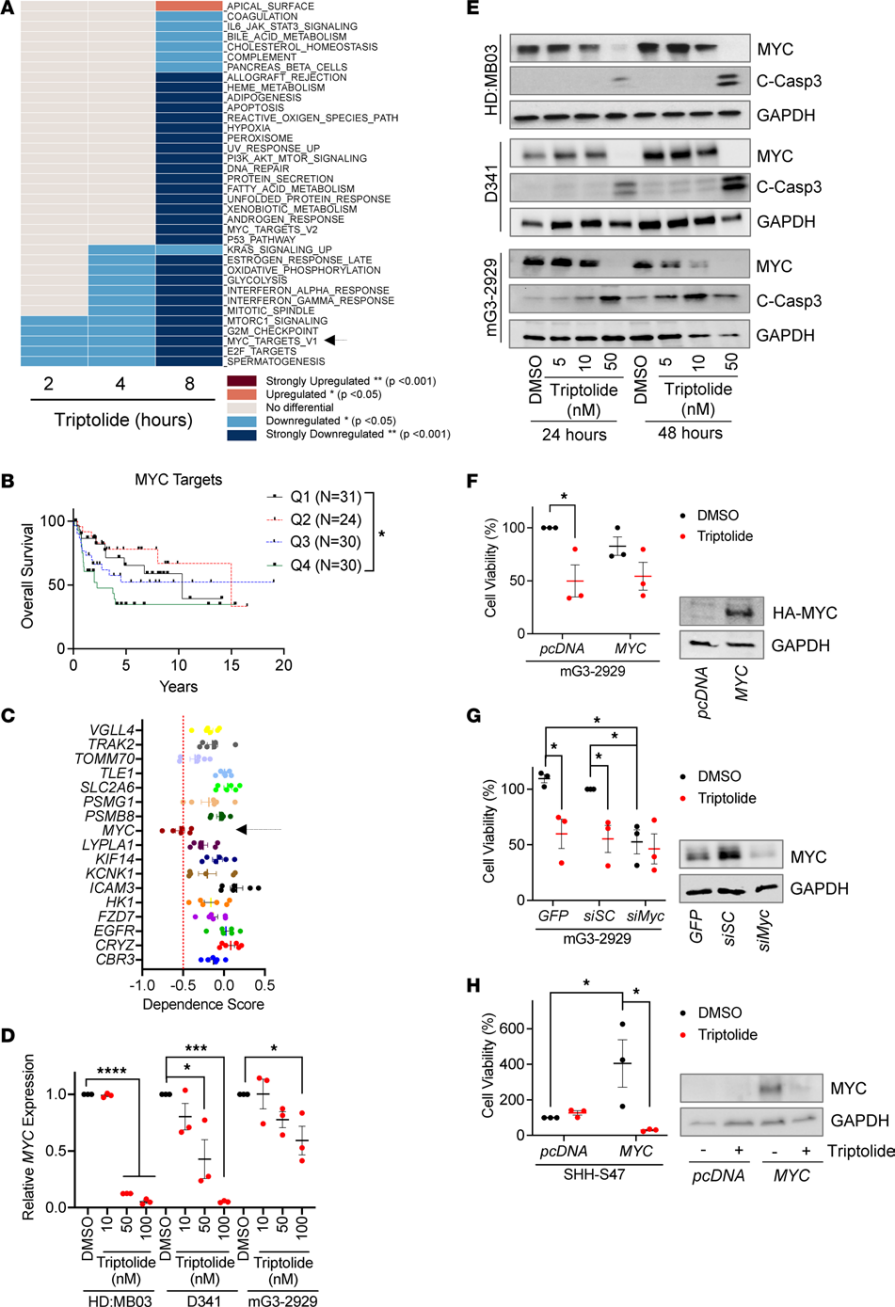
Triptolide acts on MYC to attenuate G3 MB growth. (**A**) HD:MB03 cells were treated with 10 nM triptolide, followed by RNA-seq and gene set enrichment analyses (*n* = 3). Heatmap displays triptolide-regulated gene expression hallmarks, with an arrow indicating MYC targets. (**B**) The Cavalli et al. 2017 data set was analyzed to correlate MYC targets hallmark expression with G3 MB patient survival using log-rank (Mantel-Cox) tests. (**C**) DepMap gene dependency analyses predicted G3 MB cell dependency of genes downregulated by triptolide in the L1000 triptolide transcriptional response signature in Figure 1A. Arrow highlights *MYC*. (**D**) G3 MB cells were treated with triptolide for 24 hours, and *MYC* expression was quantified by RT-qPCR (*n* = 3). Values were analyzed using 1-way ANOVA followed by Dunnett’s post hoc test. (**E**) Lysates of G3 MB cultures exposed to triptolide were immunoblotted for the indicated proteins. (**F**) mG3-2929 cells were electroporated with *HA-MYC* 72 hours prior to 50 nM triptolide treatment. Cell viability was assessed by MTT reduction 48 hours later, while MYC levels were measured by immunoblotting 72 hours after electroporation (*n* = 3). (**G**) mG3-2929 cells were transfected for 48 hours with *MYC*-targeting siRNA, scramble siRNA (*siSC*), or *GFP* control, and then treated with 50 nM triptolide. Cell viability was assessed by MTT reduction 48 hours later, and MYC levels were measured by immunoblotting 72 hours after transfection (*n* = 3). (**H**) SHH-MB47 cells received *MYC* vector via electroporation 72 hours prior to exposure to 50 nM triptolide. Cell viability was measured using CellTiter-Glo assay 48 hours later. MYC levels were assessed in similarly electroporated cells exposed to 100 nM triptolide for 16 hours (*n* = 3). Images of representative immunoblots are shown. Unless otherwise indicated, all results are presented as mean ± SEM of data normalized to DMSO, where statistical significance was assessed using 1-way ANOVA followed by Newman-Keuls post hoc test. *P < 0.05; **P < 0.01; ***P < 0.001; ****P < 0.0001.

Our data suggest that, similar to pancreatic adenocarcinoma (14), MYC levels predict triptolide response in MB. Therefore, our next step was to clarify the mechanism behind the efficacy of triptolide in this subset of tumors. Because of the pleiotropic biological effects reported for triptolide (18, 19), we performed transcriptomic analyses to identify its relevant downstream targets in G3 MB. RNA was extracted from human-derived *MYC*-amplified G3 MB cells exposed to the approximate EC_50_ of triptolide and analyzed using RNA sequencing (RNA-seq). Gene set enrichment analyses were performed to identify pathways altered in response to triptolide exposure for various time periods. We noted that 5 hallmark gene sets were significantly downregulated by triptolide within the shortest time of exposure (2 hours): mTOR complex 1 signaling, the G_2_/M cell cycle checkpoint, MYC targets, E2F targets, and spermatogenesis (Figure 3A). Importantly, additional microarray data (4) analyses showed that, of these various gene expression hallmarks, only the MYC target gene set correlated with the poor prognosis of patients with G3 MB (Figure 3B and Supplemental Figure 2, A–D). Further linking MYC with G3 MB response to triptolide, *MYC* was also included in the list of downregulated genes in the triptolide response signature shown in Figure 1, A and C (marked with an arrow). Data from the Broad Institute’s Dependency Map (DepMap) (20) also showed that of the 16 downregulated genes in the triptolide response signature, G3 MB cells exhibited the highest dependence on *MYC* (Figure 3C). Altogether, these results suggest that triptolide might act on MYC to mediate its effect in G3 MB cells.

To experimentally validate that triptolide attenuates G3 MB viability in a MYC-dependent manner, we treated 3 independent G3 MB cultures with triptolide. In these cultured cells, we observed a reduction in *MYC* gene expression (Figure 3D) and a corresponding decrease in MYC protein levels (Figure 3E), which aligned with an increase in the expression of the apoptotic marker cleaved caspase 3 (C-Casp3) (Figure 3E), suggesting a link between MYC levels and cell viability. Additionally, our gain-of-function studies demonstrated that *CMV*-driven *MYC* overexpression reduces the efficacy of triptolide in inhibiting the growth of 2 different G3 MB cultures (Figure 3F and Supplemental Figure 2E). Similarly to triptolide, knockdown of *MYC* using an siRNA-mediated approach decreased the viability of these G3 MB cultures (Figure 3G and Supplemental Figure 2F). Furthermore, in line with triptolide’s mechanism of action involving MYC, the addition of triptolide did not further decrease G3 MB cell viability in these loss-of-function analyses. Finally, to further demonstrate that triptolide works through MYC to attenuate MB growth, we transfected SHH MB cultures with a plasmid that overexpresses *MYC*. The elevated *MYC* expression resulted in faster-growing SHH MB cultures, whose MYC levels and subsequent increased viability were attenuated by triptolide (Figure 3H).

**Figure 4:**
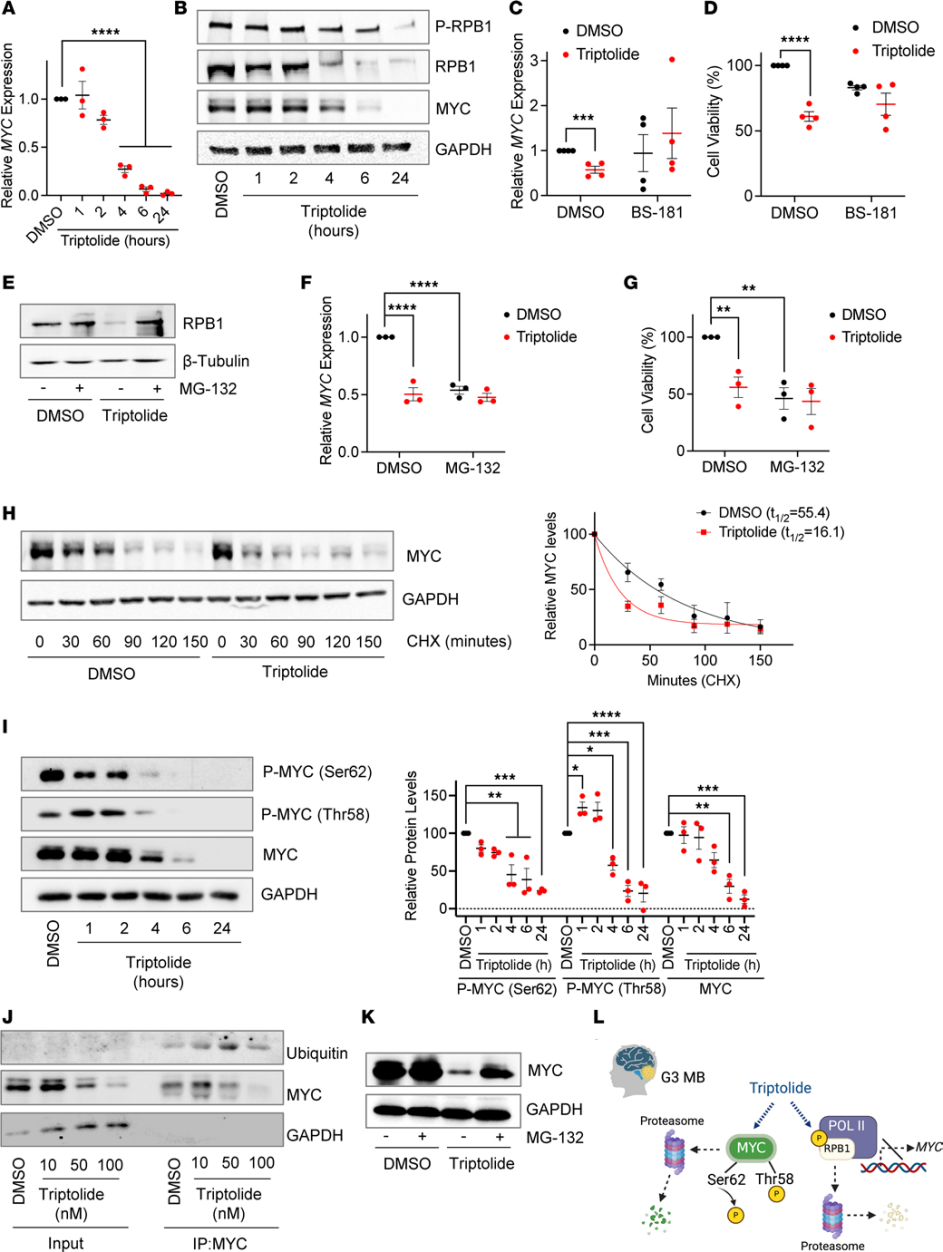
Triptolide decreases MYC levels through transcriptional and posttranslational mechanisms. (**A**) *MYC* expression in HD:MB03 cells treated with 50 nM triptolide was measured by RT-qPCR (*n* = 3) and analyzed by 1-way ANOVA followed by Dunnett’s post hoc test. (**B**) Lysates from HD:MB03 cells treated with 50 nM triptolide were immunoblotted for indicated proteins. (**C**) HD:MB03 cells were treated with BS-181 (10 μM) alone or with 50 nM triptolide for 6 hours, before determining *MYC* expression by RT-qPCR (*n* = 4). Data were analyzed using unpaired, 1-tailed Student’s *t* test. (**D**) HD:MB03 cells were treated similarly for 48 hours. Cell viability was assessed by MTT reduction (*n* = 4), and analyzed using an unpaired, 1-tailed Student’s *t* test. (**E**) HD:MB03 cells were treated with MG-132 (10 μM) for 1 hour before adding 50 nM triptolide. RPB1 levels were immunoblotted 4 hours later. (**F**) HD:MB03 cultures were similarly treated and *MYC* expression was determined by RT-qPCR 4 hours later (*n* = 3). (**G**) Cell viability was assessed by MTT reduction 48 hours after similar treatment (*n* = 3). (**H**) HD:MB03 cells were exposed to 50 nM triptolide for 2 hours before adding 25 μM CHX. MYC half-life was calculated using nonlinear regression analyses (*n* = 3). (**I**) HD:MB03 cells were treated with 50 nM triptolide. Levels of MYC and its phosphorylated forms were determined by immunoblotting (*n* = 3), and analyzed by 1-way ANOVA followed by Dunnett’s post hoc test. (**J**) HD:MB03 cells were exposed to triptolide for 4 hours before immunoprecipitating MYC. Immunoprecipitates and their input extract were immunoblotted for the indicated proteins. (**K**) HD:MB03 cells were treated with 10 μM MG-132 for 1 hour before adding 50 nM triptolide. MYC levels were immunoblotted 4 hours later. (**L**) Schematic suggesting triptolide’s mechanism of action on G3 MB. Representative immunoblots are shown. Unless otherwise indicated, statistical significance was assessed by 1-way ANOVA followed by Newman-Keuls post hoc test. *P < 0.05; **P < 0.01; ***P < 0.001; ****P < 0.0001.

We next exposed human-derived G3 MB cells to triptolide for a range of treatment times to better understand the primary mechanism of MYC regulation by triptolide. *MYC* RNA expression started to decrease after 4 hours of triptolide exposure and continued to drop thereafter (Figure 4A). It has been previously shown that triptolide acts like a super-enhancer inhibitor to block *MYC* transcription (21). To do so, triptolide was reported to phosphorylate the largest subunit of RNA polymerase II (Pol II), RNA Pol II subunit B1 (RPB1), in a cyclin-dependent kinase 7–controlled (CDK7-controlled) manner to decrease RPB1 stability (22, 23). In line with these observations, triptolide treatment of G3 MB cultures resulted in increased RPB1 phosphorylation relative to the total RPB1 levels, which decreased in parallel to those of MYC (Figure 4B). Moreover, a CDK7 inhibitor (BS-181) prevented triptolide-mediated reductions in *MYC* expression (Figure 4C) and in G3 MB cell viability (Figure 4D). Consistent with a proteasome-dependent RPB1 degradation, treating G3 MB cells with triptolide in the presence of the proteasome inhibitor MG-132 attenuated RPB1 degradation (Figure 4E). Moreover, similarly to triptolide, proteasome inhibition decreased MYC expression (Figure 4F) and G3 MB cell viability (Figure 4G), while the combined treatment of triptolide and MG-132 did not demonstrate an additive effect compared to MG-132 alone. These results suggest that, at least in part, triptolide attenuates *MYC* expression in G3 MB by reducing RPB1 stability in a proteasome-dependent manner.

Besides its role in reducing *MYC* transcription (14, 21–23), it has also been suggested that triptolide affects MYC phosphoregulation, which results in its decreased stability (24–27). As a drug affecting both *MYC* transcription and stability might explain the increased efficacy of triptolide in MYC-driven malignancies, we next studied the effect of triptolide on MYC protein stability. Experiments in which protein translation is blocked by cycloheximide (CHX) showed that triptolide reduces the MYC half-life (Figure 4H). MYC phosphorylation, such as decreased phosphorylation of Ser62 or increased phosphorylation of Thr58, is a well-known regulator of MYC stability (28, 29). We, therefore, next examined the ability of triptolide to alter the phosphorylation of MYC on these 2 sites. While a significant decrease in overall MYC protein levels was observed after 6 hours of triptolide exposure (Figure 4I), an increase in Thr58 phosphorylation was noted within the first hour, and a reduction in Ser62 phosphorylation became significant at 4 hours. As increased MYC phosphorylation on Thr58 targets it for proteasome-dependent degradation (28, 29), we next studied the involvement of the proteasome in the decrease in MYC levels induced by triptolide. Triptolide exposure increased MYC ubiquitination (Figure 4J) and proteasome inhibition prevented the decrease in MYC levels induced by triptolide (Figure 4K). Together, our results suggest that triptolide attenuates G3 MB growth by both decreasing *MYC* transcription and affecting its stability in a proteasome-dependent manner (Figure 4L).

### Triptolide attenuates G3 MB growth and metastatic spread.

**Figure 5:**
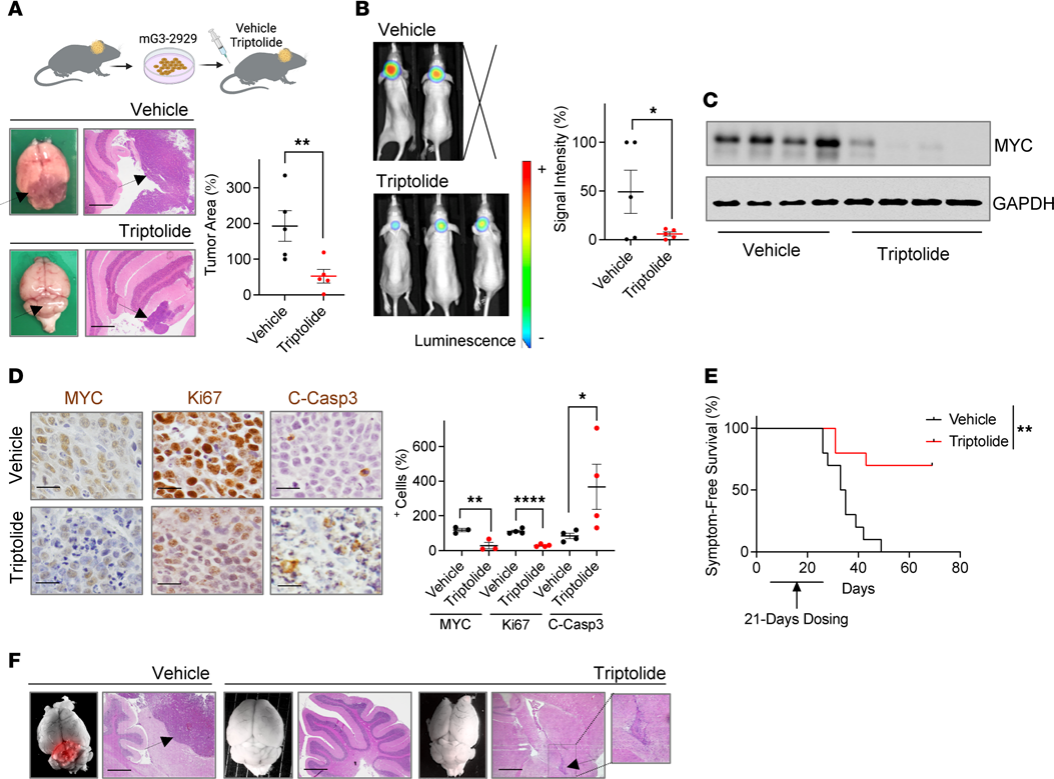
Triptolide reduces tumor growth in G3 MB mouse models. (**A**) mG3-2929 cells were implanted into mice 15 days before starting vehicle or triptolide (0.4 mg/kg, i.p., daily) dosing for 5 days. Tumor area was measured in ×2.5-magnified H&E-stained tissues (*n* = 5), and representative images of whole and H&E-stained (scale bars: 400 μm) brains are shown. (**B**) mG3-2929 cells were allowed to form tumors for 4 days prior to starting similar triptolide dosing. Tumor size was determined 12 days later by IVIS imaging (*n* = 5). (**C**) Similar cells were orthotopically implanted 15 days before starting vehicle or triptolide dosing (0.4 mg/kg, i.p., daily) for 5 days. Brain tumors were harvested and their lysates immunoblotted for MYC (*n* = 4). (**D**) Brain tumor tissues from mice similarly treated for 5 days were harvested and immunostained for the indicated proteins. Number of positive cells per field was quantified (MYC *n* = 3, Ki67/C-Casp3 *n* = 4). Representative images (scale bars: 50 μm) are shown. (**E**) mG3-2929 cells were orthotopically implanted 3 days before dosing mice with vehicle or triptolide (0.4 mg/kg, i.p., daily) for 21 days. Symptom-free survival was analyzed using log-rank (Mantel-Cox) tests (*n* = 10). (**F**) Displayed are whole and H&E-stained (scale bars: 400 μm) brains from a symptomatic mouse in the vehicle cohort, along with 2 representative animals that remained asymptomatic 20 days after the last vehicle-treated mouse was euthanatized. RFP signal and arrows indicate tumor presence. In all cases, brains were harvested 6 hours after the last injection. Unless otherwise indicated, all results are presented as mean ± SEM of data normalized to 1 vehicle-treated animal. Statistical significance, unless otherwise specified, was assessed using an unpaired, 1-tailed Student’s *t* test. *P < 0.05; **P < 0.01; ****P < 0.0001.

Triptolide crosses the blood-brain barrier and accumulates in the brain at levels comparable to those found in various organs (30), including those currently undergoing clinical evaluation for its derivatives (11, 31). Thus, we next investigated whether triptolide would not only reduce MYC levels, but also tumor burden in an orthotopic mouse model of MB. Similar to our ex vivo observations, mG3-2929–derived tumors in mice exposed to triptolide were smaller (Figure 5, A and B), showed reduced MYC levels (Figure 5C), and contained a lower number of MYC-expressing cells (Figure 5D and Supplemental Figure 3A). G3 MB tumors in triptolide-treated mice also exhibited a lower proliferative index and increased number of apoptotic cells compared with the vehicle-dosed controls (Figure 5D and Supplemental Figure 3A). Importantly, treatment with triptolide significantly prolonged the symptom-free survival of mice harboring similar *Myc*-overexpressing tumors (Figure 5E). We next determined whether the triptolide-treated mice, which remained asymptomatic 20 days after the last vehicle-treated animal developed MB symptoms, had any detectable tumor mass. Further supporting the efficacy of triptolide in abrogating G3 MB growth, signs of residual disease were found only in the brains of 2 out of 7 of these mice (Figure 5F). Collectively, our data demonstrate that triptolide decreases MYC levels in vivo*,* resulting in a decline in tumor growth and an overall increase in survival of mice harboring *Myc*-amplified G3 MB.

**Figure 6:**
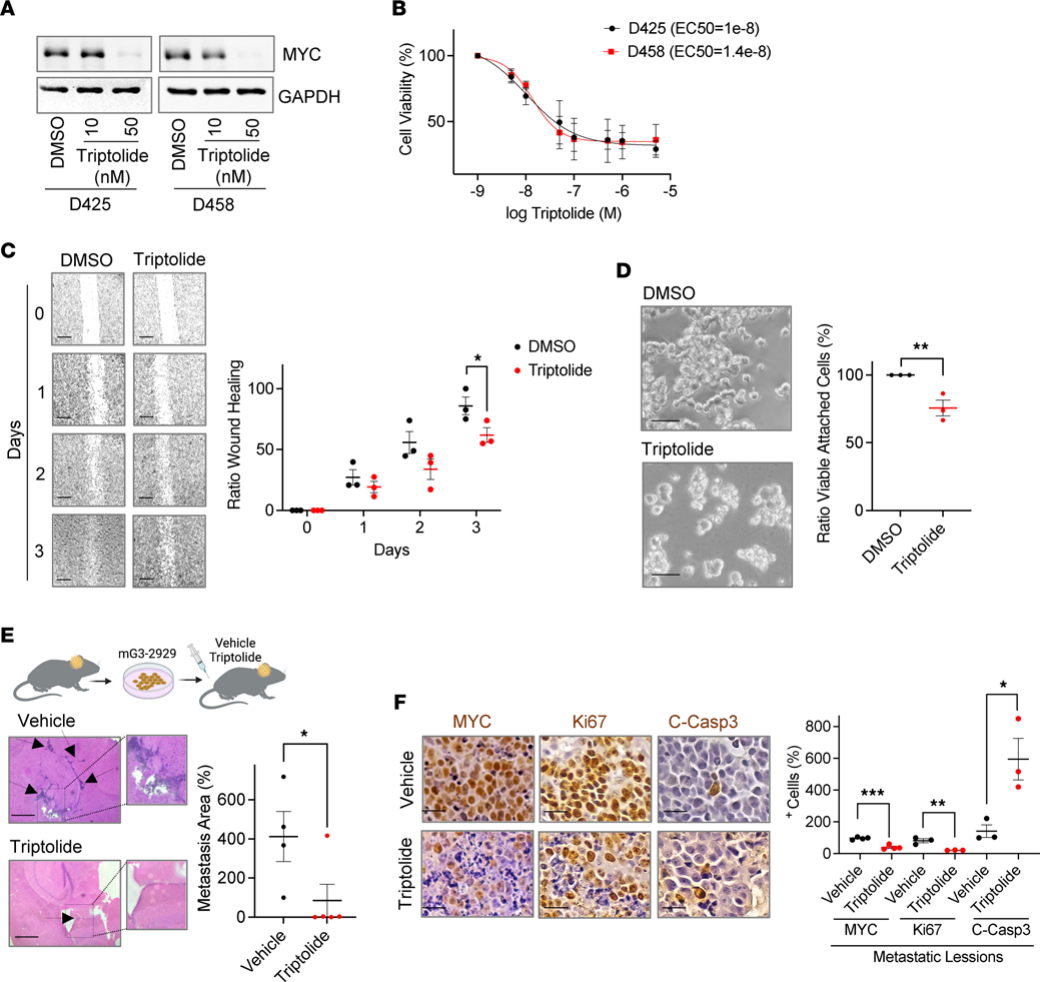
Triptolide attenuates G3 MB metastatic dissemination. (**A**) MYC levels were determined in D425 (primary) and D458 (metastatic) tumor cells treated with triptolide for 16 hours. Representative immunoblots are shown. (**B**) Similar cells were exposed to triptolide for 48 hours and cell viability was determined by MTT reduction. Nonlinear regression analyses on the mean ± SEM of data normalized to DMSO were performed (*n* = 3). (**C**) Wound healing assays were performed in HD:MB03 cultures treated with 10 nM triptolide. A wound healing ratio was calculated relative to initial measurement. Mean ± SEM of the wound ratio per day (*n* = 3) and representative images (scale bars: 400 μm) are shown. (**D**) mG3-2929 sphere cultures exposed to 10 nM triptolide were plated on poly-D-lysine–coated wells for 24 hours. The ratio of viable attached cells, as determined by MTT reduction, to total viable cells was calculated. Mean ± SEM of data normalized to DMSO was plotted (*n* = 3). Representative images (scale bars: 50 μm) are shown. (**E**) mG3-2929 cells were implanted into mice 15 days before starting vehicle or triptolide (0.4 mg/kg, i.p., daily) dosing for 5 days. Metastatic lesions outside of the posterior fossa were quantified in ×2.5-magnified H&E-stained brains (vehicle *n* = 4, triptolide *n* = 5). Detail of metastatic lesions in representative images is shown. Arrows indicate tumor presence. (**F**) Mice harboring mG3-2929 tumors were similarly treated with vehicle or triptolide for 5 days, before quantifying numbers of positive cells in metastatic regions by IHC analyses (MYC *n* = 4, Ki67/C-Casp3 *n* = 3). Representative images (scale bars: 50 μm) are shown. In all cases, brains were harvested 6 hours after the last injection. Unless otherwise indicated, all results are presented as mean ± SEM of data normalized to 1 vehicle-treated animal. Statistical significance was assessed using an unpaired, 1-tailed Student’s *t* test. *P < 0.05; **P < 0.01; ***P < 0.001.

As G3 MB is frequently metastatic at diagnosis (4–6), we next determined whether triptolide could attenuate the leptomeningeal spread of this subset of tumors. Triptolide similarly attenuated MYC levels (Figure 6A) and reduced the viability (Figure 6B) of cell cultures grown from a primary human G3 MB (D425) and from a metastatic disease (D458) found in the same patient. Moreover, triptolide also showed efficacy in attenuating cell migration (Figure 6C) and invasion (Figure 6D) in G3 MB cultures. In line with these ex vivo observations, mice implanted with mouse-derived G3 MB tumors and treated with triptolide exhibited smaller metastatic lesions in the ventricular region and in the subarachnoid space (Figure 6E). Moreover, similar to the primary tumors located in the posterior fossa, the metastatic lesions observed in mice treated with triptolide had lower numbers of MYC-expressing cells, a reduced Ki67 index, and increased apoptosis (Figure 6F and Supplemental Figure 3B).

### The triptolide prodrug Minnelide shows efficacy in preclinical MB models.

**Figure 7:**
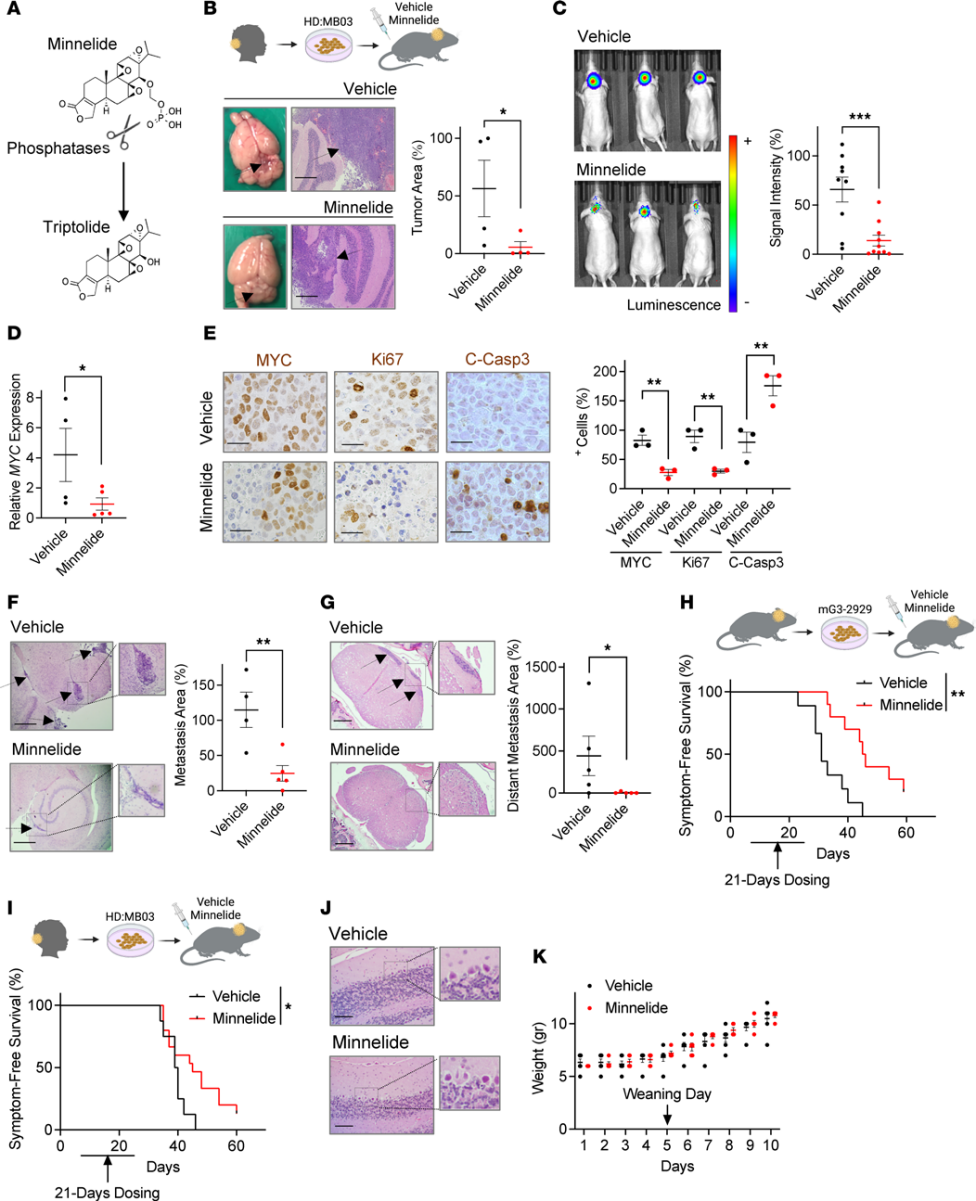
The triptolide prodrug, Minnelide, attenuates G3 MB growth. (**A**) Schematic showing Minnelide hydrolyzation into active triptolide. (**B**) Mice were implanted with HD:MB03 cells 15 days before being treated with either vehicle or Minnelide (0.4 mg/kg, i.p., daily) for 4 days (*n* = 4). (**C**) HD:MB03 cells were allowed to form orthotopic tumors for 3 days before similarly dosing mice for 7 days. Tumor size was quantified by IVIS imaging (vehicle *n* = 9, Minnelide *n* = 10). (**D**) HD:MB03 cells were allowed to form orthotopic tumors for 15 days before similarly dosing mice for 4 days. *MYC* expression in harvested brains was determined by RT-qPCR (vehicle *n* = 4, Minnelide *n* = 5). (**E**) Immunostaining for indicated proteins in brain tumors from similar mice (*n* = 3). (**F**) Measurement of metastatic lesions located outside of the posterior fossa of similar mice (vehicle *n* = 4, Minnelide *n* = 5). (**G**) Tumors located in the spinal cord of mice similarly dosed for 21 days were measured (*n* = 5). (**H**) mG3-2929 cells were implanted 3 days before similar dosing, and symptom-free survival was determined using log-rank (Mantel-Cox) tests (vehicle *n* = 9, Minnelide *n* = 10). (**I**) Symptom-free survival was determined using log-rank (Mantel-Cox) tests in similarly dosed mice but harboring HD:MB03 tumors (vehicle *n* = 8, Minnelide *n* = 15). (**J**) Mice were similarly treated for 21 days before examining cerebellar tissues (*n* = 5). (**K**) Displayed are body weights of 2-week-old wild-type mice similarly dosed for 10 days (vehicle *n* = 5, Minnelide *n* = 6). All tumor area measurements were performed in ×2.5-magnified H&E-stained slides. All images are representative. Arrows indicate tumor presence. All tissues were harvested 6 hours after the last injection. Results are presented as mean ± SEM of data normalized to 1 vehicle-treated animal. Scale bars: 400 μm (**B**, **F**, **G**, and **J**) and 50 μm (**E**). Statistical significance was assessed using unpaired, 1-tailed Student’s *t* test. *P < 0.05; **P < 0.01; ***P < 0.001.

Given the observed effects of triptolide, we next determined the efficacy of its prodrug in clinical development, Minnelide (11, 31), in G3 MB models. Minnelide is an inactive water-soluble derivative of triptolide that gets converted into active triptolide by phosphatases present in all body tissues, including blood (Figure 7A). Similarly to triptolide, upon phosphatase-mediated activation, Minnelide attenuated the growth of G3 MB cultures (Supplemental Figure 4A), reduced MYC levels, and increased the cleavage of the apoptosis effector caspase, Casp3 (Supplemental Figure 4B). Given the rapid conversion of Minnelide into active triptolide in vivo (12) and its documented accumulation in brain tissues (30), we next assessed the efficacy of Minnelide in orthotopic mouse MB models. Minnelide was administered in these experiments at previously reported dosages (12, 32–35). Mice orthotopically implanted with luciferase-expressing G3 MB cells (HD:MB03) were exposed to vehicle or Minnelide. Tumor size was subsequently determined by measuring the tumor area in H&E-stained tissues (Figure 7B) and by quantifying luciferase activity (Figure 7C). Similar to triptolide, Minnelide-treated mice exhibited smaller tumors than those exposed to vehicle. *MYC* expression (Figure 7D) and the number of cells expressing MYC (Figure 7E and Supplemental Figure 4C) were also reduced in tumors from Minnelide-dosed mice, and were therefore less proliferative and more apoptotic than those treated with vehicle (Figure 7E and Supplemental Figure 4C). Moreover, mice exposed to Minnelide displayed smaller metastatic lesions in brain (Figure 7F) and spinal cord (Figure 7G) tissues. Importantly, Minnelide-treated mice harboring either mouse- (Figure 7H) or human-derived (Figure 7I) G3 tumors had longer symptom-free survival compared with those dosed with vehicle. While Minnelide and triptolide demonstrated similar EC_50_ values ex vivo, differences in in vivo efficacy were noted. These variations could arise in part from the administration of a lower effective dose of Minnelide, although the regimen used was supported by previous Minnelide preclinical studies (12, 32–35). Due to differences in molecular weights, 0.7 molar equivalents of Minnelide were administered relative to triptolide when 0.4 mg/kg dose levels were administered for each agent. Therefore, mice given Minnelide were exposed to less active drug, likely contributing to variation in efficacy.

Considering clinical observations of Minnelide inducing reversible cerebellar toxicity (36), we also examined the integrity of cerebellar tissues in tumor-bearing mice treated with Minnelide. Examination of the cerebellum from Minnelide-exposed mice by a board-certified pathologist revealed normal cortical architecture and indistinguishable cytologic features compared to vehicle-treated animals (Figure 7J). Specifically, Purkinje and granule neurons showed no differences in appearance, while no signs of neuroinflammation were observed, except at the interfaces with the tumor. Moreover, demonstrating its tolerability in young animals, no noticeable differences were observed in the overall ability to gain weight of mice whose Minnelide dosing started 5 days prior to weaning age (Figure 7K).

**Figure 8:**
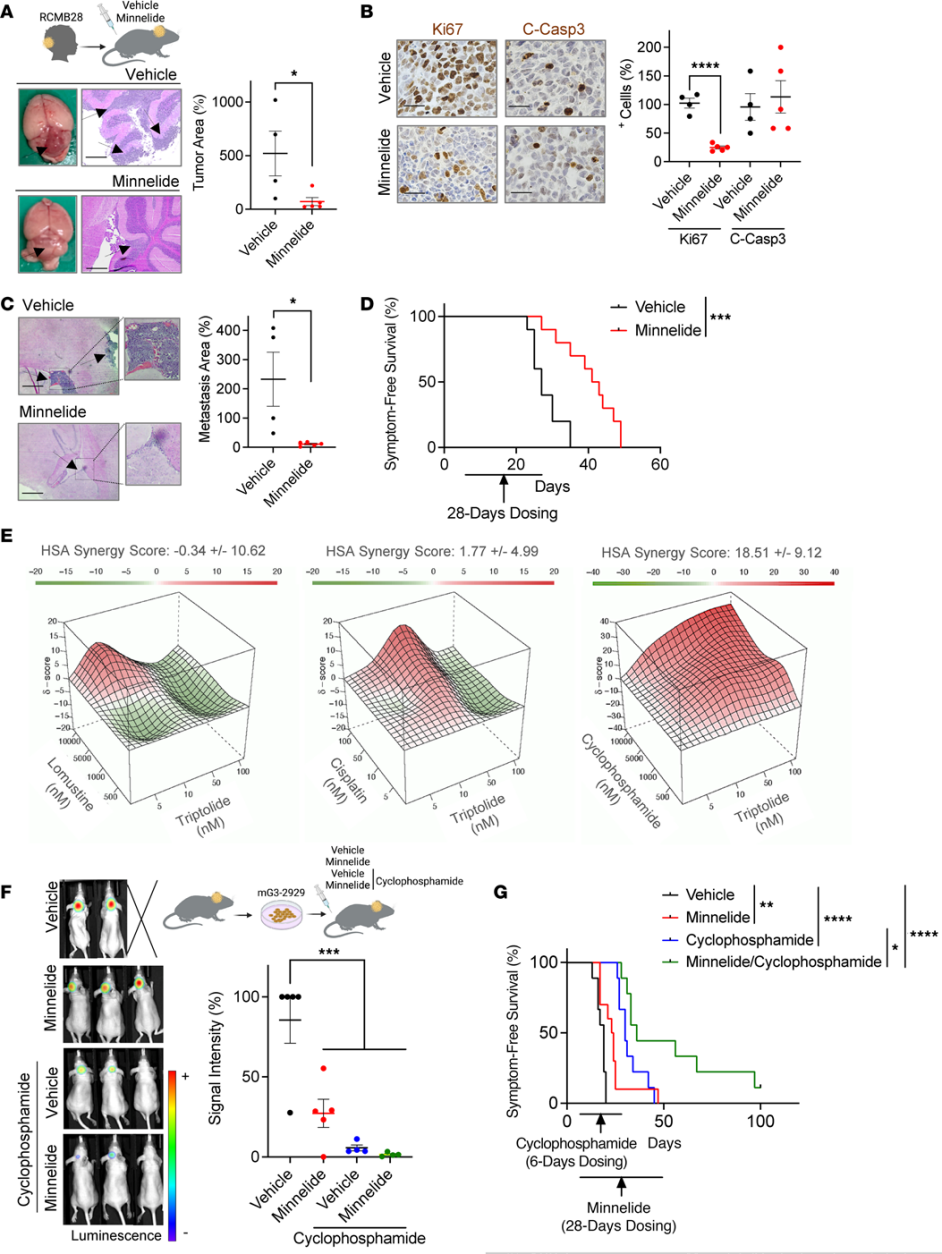
Minnelide shows translational potential. (**A**) RCMB28 PDOX cells were implanted into mice 3 weeks before vehicle or Minnelide (0.4 mg/kg, i.p., daily) dosing. Five days later, tumor areas in their brains were measured (vehicle *n* = 4, Minnelide *n* = 5). (**B**) Numbers of Ki67- and C-Casp3–positive cells in brains from similarly treated mice were quantified by IHC analyses (vehicle *n* = 4, Minnelide *n* = 5). (**C**) Metastatic lesions of mice harboring RCMB28-derived tumors and similarly dosed were measured (vehicle *n* = 4, Minnelide *n* = 5). (**D**) RCMB28 were orthotopically implanted 3 days before starting similar vehicle or Minnelide dosing. Symptom-free survival was determined and analyzed using log-rank (Mantel-Cox) tests (*n* = 10). (**E**) mG3-2929 cultures were exposed to triptolide alone or in combination with indicated compounds. Cell viability was determined by MTT reduction, and SynergyFinder was used to generate 3D surface plots and obtain synergy scores (cisplatin *n* = 4, lomustine *n* = 3, cyclophosphamide *n* = 5). An HSA score of greater than 10 indicates synergy. (**F**) Mice were implanted with mG3-2929 cells 8 days before daily dosing with cyclophosphamide (65 mg/kg, i.p.), alone or with Minnelide (0.4 mg/kg, i.p.). Tumor burden was determined by IVIS imaging 8 days later (vehicle/Minnelide *n* = 5, cyclophosphamide/combination *n* = 4), and analyzed using 1-way ANOVA followed by Newman-Keuls post hoc test. (**G**) mG3-2929 were allowed to form tumors for 8 days before starting similar dosing. Symptom-free survival data were analyzed using a log-rank (Mantel-Cox) test (vehicle/cyclophosphamide/combination *n* = 9, Minnelide *n* = 10). All tumor area measurements were performed in ×2.5-magnified H&E-stained slides. All images are representative. Arrows indicate tumor presence. All tissues were harvested 6 hours after the last injection. Results are presented as mean ± SEM of data normalized to 1 vehicle-treated animal. Scale bars: 400 μm (**A** and **C**) and 50 μm (**B**). Statistical significance was assessed using unpaired, 1-tailed Student’s *t* test. *P < 0.05; **P < 0.01; ***P < 0.001; ****P < 0.0001.

We next evaluated the efficacy of Minnelide in a patient-derived orthotopic xenograft (PDOX) model (RCMB28) expanded from a patient harboring a *MYC*-amplified G3 MB (37), rather than the previous mouse models in which tumors were derived from G3 MB cells in culture. Treatment of mice bearing RCMB28 PDOX tumors, which had never been grown in culture, with Minnelide resulted in a reduction in both tumor size (Figure 8A) and tumor cell proliferation (Figure 8B and Supplemental Figure 4B). Furthermore, highlighting its predominant impact on the proliferation of this tumor model, Minnelide did not influence Casp3 cleavage (Figure 8B and Supplemental Figure 4D). Importantly, and in line with our data on tumors grown from G3 MB cultures, Minnelide also attenuated metastatic spread (Figure 8C) and increased survival (Figure 8D) of mice carrying this PDOX G3 MB model. Together, these results highlight the efficacy of Minnelide in attenuating MYC-driven G3 MB growth.

To further explore the translational potential of our work, we investigated whether Minnelide could enhance the effectiveness of standard-of-care chemotherapy. Due to the poor outcome in patients with these tumors, a disease model (mG3-2929) simulating 2 prevalent genetic events found in recurrent G3 MB, *MYC* amplifications and *TP53* mutations (7, 8), was selected for these studies. Synergy studies involved treating mG3-2929 MB cells with increasing concentrations of triptolide and chemotherapeutics included in treatment protocols for patients with MB such as lomustine, cisplatin, and cyclophosphamide (16). Among these compounds, only the alkylating agent cyclophosphamide exhibited a synergy score indicating the potential for these 2 compounds to act synergistically (Figure 8E and Supplemental Figure 5). In our evaluation of this drug combination in mice with similar disease, a short course of Minnelide dosing did not significantly increase the efficacy of cyclophosphamide in reducing tumor burden (Figure 8F). However, extended Minnelide administration improved survival rates (Figure 8G) in mice treated with both compounds. Together, our results support the translation of Minnelide for the treatment of G3 MB patients, including those with highly aggressive recurrent tumors.

## Discussion

Although the efficacy of triptolide in attenuating the growth of distinct malignancies has been previously reported (11, 18, 19, 31), its efficacy against pediatric brain tumors such as MB had not yet been explored. Using a bioinformatic approach, we predicted the sensitivity of G3 MB tumors to triptolide. Consistent with this prediction, triptolide attenuated the growth of G3 MB at doses significantly lower than those required for SHH MB cells. Our results showed that triptolide acts on MYC to attenuate growth in G3 MB cultures. Moreover, triptolide reduced MYC levels in these cultures by affecting both *MYC* transcription and overall MYC protein stability. Importantly, triptolide and Minnelide were both able to attenuate the growth of primary and metastatic lesions in various G3 MB mouse models, subsequently increased the survival of tumor-bearing mice, and they did so in a MYC-dependent manner. Thus, our results highlight the potential for Minnelide, which has previously been found to be safely tolerated in clinical trials (13), for the treatment of patients with metastatic high-risk G3 MB. Further supporting such translational potential, Minnelide also increased the efficacy of standard-of-care chemotherapy in a G3 MB mouse model.

Previous observations in targeting pancreatic ductal adenocarcinoma suggest that MYC levels can be used to predict the response to triptolide, with tumor cultures harboring the highest MYC levels being more sensitive (14). Accordingly, we show that G3 MB tissues harboring *MYC* amplifications are more likely to respond to triptolide. In addition to the correlation between MYC levels and MB response, and in line with previous findings (14, 21, 22, 27, 38), our results suggest that triptolide regulates the levels of this oncogene to attenuate MB growth. Triptolide has been proposed to be a super-enhancer inhibitor that controls the expression of a range of oncogenes (21). This effect is achieved through its binding to the helicase xeroderma pigmentosum type B (XPB), observed in both mammalian (39, 40) and fly (41) models, leading to the subsequent degradation of RPB1 (22, 23). The loss of RPB1 results in an overall reduction in RNA Pol II–mediated gene transcription (14, 23, 40), especially noticeable in short-half-lived transcripts such as *MYC* (42). In line with this mechanism, triptolide reduced *MYC* expression in G3 MB cultures by triggering the degradation of RPB1. CDK7, whose role in the growth of *MYC*-amplified MB was previously described (43), facilitated RPB1 degradation by phosphorylating it, a process that leads to its proteasomal degradation (23). Despite its impact on *MYC* expression, previous studies have suggested that triptolide could also act as a MYC degrader (24–27). Our time-course experiments performed in the presence of CHX also support these findings, revealing that triptolide decreases MYC half-life in G3 MB. This effect is likely due to increased MYC phosphorylation on Thr58, a modification known to target MYC for proteasome-dependent degradation (28, 29). Collectively, these data suggest that triptolide reduces both *MYC* expression and MYC stability, explaining the heightened sensitivity of *MYC*-amplified malignancies, including G3 MB, to this compound.

The prognosis of MB patients is dependent on the various molecular drivers within each subgroup. While most children with WNT MB have an excellent prognosis, G3 MB patients harboring *MYC* amplifications exhibit the lowest 5-year overall survival of all MB subgroups (4). It is therefore imperative to find better treatments to improve the outcomes of these latter patients. In line with this therapeutic need, we demonstrated the efficacy of triptolide and its clinically relevant derivative, Minnelide, in reducing the growth of MYC-driven MB. Previous studies in MB have indicated that primary and metastatic lesions may not be identical (44). Despite these described differences, Minnelide demonstrated effectiveness in reducing the leptomeningeal spread of *MYC*-amplified MB, a characteristic feature present in nearly half of these tumors (4–6) and a key factor contributing to their unfavorable prognosis (15). Furthermore, *MYC* amplifications, along with mutations in the tumor suppressor *TP53*, are frequently observed in relapsed G3 MB (7, 8). These recurrent patients face a very poor outcome (7, 8), highlighting the need to develop tailored therapies for them. Addressing this demand, we showed that Minnelide not only abrogates the growth of tumors that harbor *TP53* mutations and *MYC* amplification/overexpression, but also enhances their response to standard-of-care chemotherapy. Despite the overall efficacy of Minnelide alone as well as in combination with cyclophosphamide, most of the animals eventually succumbed to their disease. Future studies should be performed to determine whether tumors regrew due to insufficient dosing or to the clonal expansion of molecularly or genetically distinct tumor cells underlying the development of treatment-resistant relapsed disease. In summary, our results establish the efficacy of Minnelide for the treatment of primary and recurrent *MYC*-amplified G3 MB, thereby supporting the inclusion of these subsets of high-risk patients in ongoing or future Minnelide clinical trials.

## Methods

### Sex as a biological variable.

Sex was not considered as a biological variable and therefore in vivo studies were not powered to detect sex-related differences in efficacy.

### Cell culture.

HD:MB03 cells (Leibniz Institute DSMZ) isolated from a 3-year-old patient with metastatic *MYC*-amplified G3 MB (45) were maintained in RPMI-1640 media (Gibco) containing 10% fetal bovine serum (FBS, Atlanta Biologicals), 1% nonessential amino acids (Gibco), and penicillin-streptomycin (Invitrogen). HD:MB03 cells engineered to express luciferase were a gift from Noriyuki Kasahara (UCSF, San Francisco, California, USA). The *MYC*-amplified G3 MB cell line D341 (ATCC) from a 3.5-year-old patient (46) was maintained in Eagle’s minimal essential medium from Gibco, 20% FBS, and penicillin-streptomycin. Granule neuron progenitors purified from *Ink4c*-null (47); *Trp53*-null (48) mice and transduced with a retrovirus coexpressing mouse *Myc* and red fluorescent protein (*RFP*) were implanted into the cortex of *CD1-Foxn1^nu^* mice (Charles River Laboratories), as previously described (49). Two independent mouse MB cultures (mG3-2929 and mG3-2922) were obtained and propagated ex vivo in Neurobasal-A serum-free media containing GlutaMax (Gibco), B27, N2, penicillin-streptomycin (all from Invitrogen), and supplemented with epidermal growth factor (EGF, 40 ng/mL) and fibroblast growth factor (FGF, 40 ng/mL), both from Prepotech. D425 cells (Invitrogen) isolated from a 6-year-old patient with G3 MB and D458 cells isolated from the leptomeningeal spread of this same patient (50) (a gift from Darell Bigner (Duke University Medical Center, Durham, North Carolina, USA), were cultured in high-glucose Dulbecco’s modified Eagle medium (DMEM; Gibco) supplemented with 10% heat-inactivated FBS (Atlanta Biologicals), GlutaMax, and penicillin-streptomycin. The SHH subgroup Daoy cells (ATCC) from a 4-year-old patient (51, 52) were cultured in DMEM, 10% FBS, and penicillin-streptomycin. Smoothened inhibitor–sensitive SHH-S47 and SHH-S1 were derived from spontaneous MB developed in *Ptch1-LacZ* (*Ptch1^tm1Mps^*/J) or *Ptch1-LacZ; Trp53*-KO (B6.129S2-*Trp53^tm1Tyj^*/7) mice (both from the Jackson Laboratory), respectively, and cultured in DMEM/F12 (Gibco), B27, and penicillin-streptomycin (53, 54). Triptolide and CHX were acquired from Millipore-Sigma, while the rest of the drugs used in cell cultures were from Selleckchem.

### Mouse studies.

For studies using mG3-2929 and HD:MB03, 3 × 10^5^ or 6 × 10^5^ viable cells, respectively, were implanted into 5- to 8-week-old *CD1-Foxn1^nu^* mice. Viable cells (1 × 10^6^) from the PDOX RCMB28 (GEO GSM4575704) isolated from a 15-year-old patient with G3 MB, courtesy of Robert Wechsler-Reya (Sanford Burnham Prebys, La Jolla, California, USA), were implanted into 5- to 8-week-old NOD.SCID mice (NOD.CB17-*Prkdc^scid^*/NCrHsd) from Envigo. The number of viable cells to implant was determined by a trypan blue exclusion assay in a TC20 cell counter (Bio-Rad). In all cases, cells were resuspended in 3 μL Neurobasal-A serum-free media and orthotopically implanted into the cerebellum using the coordinates 2 mm down lambda, 2 mm right of the midline suture, and 2 mm deep (53). Mice were dosed with 0.4 mg/kg (1.11 μmol/kg) of triptolide (Selleckchem) in 50 μL 100% DMSO, resulting in a final concentration of DMSO of 0.124 mL/kg per mouse — a concentration significantly lower than its reported LD_50_ (55). Minnelide was either provided by Sulagna Banerjee (University of Miami, Miami, Florida) or purchased from MedchemExpress, and dosed at 0.4 mg/kg (0.77 μmol/kg) in PBS. Administration of triptolide and Minnelide was performed daily via intraperitoneal (i.p.) injection. For in vivo drug combination studies, cyclophosphamide (Millipore-Sigma) and Minnelide were dissolved in PBS and administered daily at 65 mg/kg and 0.4 mg/kg, respectively.

For immunohistochemistry (IHC) and immunoblotting studies, tumors were allowed to grow until a fully established disease was predicted (2 weeks for mG3-2929 and HD:MB03 cells, and 3 weeks for RCMB18 PDOX cells) before starting the dosing regimen. For in vivo imaging (IVIS) studies, luciferase-expressing HD:MB03 and mG3-2929 cells were orthotopically implanted and allowed to grow for 3 to 4 days in all cases, except in combination therapy studies, where tumors were allowed to grow for 8 days before starting drug dosing. For imaging, D-luciferin (Perkin Elmer) was administered i.p. (150 mg/kg) 10 minutes before imaging in a Caliper/Xenogen IVIS Spectrum, as previously described (53). Mice that died before imaging were assigned the readout corresponding to the animal with the highest signal in the experiment. For symptom-free survival studies, tumors were allowed to grow for 3 to 4 days before starting the dosing regimen, except for the combination therapy studies in which tumors were allowed to grow for up to 8 days. Mice were sacrificed upon developing signs of brain tumor growth, including a dome-shaped cranium indicative of hydrocephalus, head tilting, hunching, circling, hemineglect, loss of weight, or ataxia. The presence of a brain tumor was confirmed by H&E staining, and by IVIS imaging for mice implanted with luciferase-expressing HD:MB03 or mG3-2929 cells, or by imaging for RFP signal on a Nikon SMZ18 fluorescence stereoscope in brains implanted with mG3-2929 cells.

### IHC.

After in vivo treatment, brains were harvested, fixed for 48 hours in 10% neutral buffered formalin (VWR), and paraffin embedded. Spines were similarly fixed for 3 days and then decalcified in Formical-2000 (Statlab) before being embedded in paraffin. Antigen retrieval was performed by steaming tissue sections on glass slides in citrate buffer for 30 minutes prior to staining. Antibodies that detect C-Casp3 were obtained from Cell Signaling Technology, while those detecting Ki67 and MYC were from Abcam (Supplemental Table 1), and all were used according to the manufacturer’s recommendations. HRP SignalStain Boost Detection (Cell Signaling Technology) and Diaminobenzidine (DAKO) reagents were applied for antigen detection and counterstained with hematoxylin (Sigma-Aldrich). IHC images were obtained using a Leica DM2000 LED microscope and high-magnification images were analyzed using Nikon Imaging Software. The number of positive cells in tumor tissues was assessed using a blinded visual score of stained tissues.

### Cell and molecular biology.

Cell viability was monitored by the reduction of 3-(4,5-dimethyl-2-thiazolyl)-2,5-diphenyl-2*H*-tetrazolium bromide (MTT) to formazan as described previously (56), or by performing a trypan blue exclusion assay (Bio-Rad) or a CellTiter-Glo assay (Promega) according to the manufacturers’ recommendations. For cell proliferation assays, cells were first incubated in the presence of 5-ethynyl-2′-deoxyuridine (EdU) for 1 hour, followed by completion of the EdU detection protocol using the Cell Proliferation/DNA Synthesis Kit (Biovision) according to the manufacturer’s instructions. Subsequently, the cells were stained for C-Casp3 using antibodies from Cell Signaling Technology (Supplemental Table 1). MB spheres were immobilized onto Superfrost glass slides using a cyto-spin centrifuge (Thermo Fisher Scientific) prior to staining, while chamber slides (Millipore) were used for cultures growing as monolayers. High-magnification images of staining were taken using a Revolve fluorescence microscope (Echo) and the number of positive cells per field was assessed using ImageJ (NIH) and validated using a blinded visual score. The wound healing analyses were conducted in 80%–90% confluent monolayer cultures, where scratches were made using a 200 μL tip. The size of the wound was measured daily under a Revolve microscope, with measurements from the well edges excluded. The ratio of the daily wound measurement to the measurement taken before starting triptolide treatment was calculated. For invasion assays, sphere cultures were allowed to adhere to a poly-D-lysine–coated matrix (57). The ratio of viable cells that invaded this matrix was calculated by dividing the number of viable attached cells, as assessed through an MTT reduction assay, by the total viable cells per well. For knockdown studies, Lipofectamine 2000 (Invitrogen) was used to transfect 3 × 10^5^ cells with 25 nM siGENOME from Dharmacon consisting of a pool of 4 siRNA sequences. Studies in which *MYC* was overexpressed in MB sphere cultures were performed by electroporating either 2 × 10^6^ mG3-2929 cells or 4 × 10^6^ SHH-MB47 with 5 mg DNA using a P3 Primary Cell 4D-Nucleofector X kit in a 4D Nucleofector System (Lonza). Lipofectamine 2000 was used according to manufacturer recommendations to transfect similar vectors in HD:MB03 cells. DNA constructs used to complete these gain-of-function studies — *pcDNA3*.1 *acGFP* (catalog 128047), *pcDNA3*.1 *MYC* (catalog 176045) (58), and *pCDNA3-HA-HA-CMYC* (catalog 74164) (59) — were all obtained from Addgene.

Total RNA was TRIzol (Invitrogen) extracted and the expression of the indicated genes was analyzed by quantitative real-time PCR (RT-qPCR) using TaqMan probes as per the manufacturer’s recommendations (Invitrogen). RT-qPCR data were normalized to that of the reference genes glyceraldehyde-3-phosphate dehydrogenase (*GAPDH*) or TATA-box binding protein (*TBP*). Radio-Immunoprecipitation Assay (RIPA) buffer (Thermo Fisher Scientific) supplemented with the HALT protease inhibitor cocktail (Thermo Fisher Scientific) was used for protein extraction and the levels of the indicated proteins were determined by immunoblotting. All primary and secondary antibodies used for immunoblotting were purchased from Cell Signaling Technology and used according to the manufacturer’s recommendations (Supplemental Table 1). MYC was immunoprecipitated from cells lysed in Cell Signaling Technology lysis buffer lacking sodium dodecyl sulfate (SDS) using anti-MYC antibodies from Abcam and True-Blot beads (Rockland). Immunoprecipitated proteins were resolved via SDS–polyacrylamide gel electrophoresis (SDS-PAGE) and immunoblotted using anti-ubiquitin or anti-MYC antibodies from Cell Signaling Technology (Supplemental Table 1), and True-Blot secondary antibodies (Rockland) to prevent denatured/reduced immunoglobulin G (IgG) detection. MYC half-life in triptolide-treated cells was determined upon adding CHX and performing nonlinear regression (1-phase decay) analyses on MYC levels normalized to those of GAPDH.

### Bioinformatic analyses.

GSE37418 microarray expression data of 76 patient-derived MB samples spanning the different subtypes (Robinson et al., 2012 data set) (60) was downloaded from the St. Jude Cloud (61) and analyzed to predict the response to triptolide. MB subgroup signatures were generated by calculating the log_2_(fold change) of median expression within each subgroup to the median expression of all tumors within the data set for each gene. MB subgroup markers were identified by normalizing the average expression values to the mean of all tumors from the 4 subgroups within the data set. These differentiating signatures were then used to determine a predictive score of the effect that a compound would have on each subgroup. A triptolide response signature was generated using the LINCS L1000 data set (17), as previously described (62). Briefly, level 5 processed data from 18 cell lines treated with triptolide were downloaded from the LINCS data portal. Downloaded *z* scores were opened in R using the package cmapR, and were filtered for 24-hour treatment samples only (https://github.com/cmap/cmapR). Genes retained within the triptolide signature were those whose expression changed in the same direction (up- or downregulated) in response to triptolide treatment in at least 30% of the cell lines tested. The signature itself is comprised of the mean *z* score values for these genes relative to vehicle-treated control cells of all 18 cell lines (Supplemental Figure 6). Spearman’s correlations were calculated between the triptolide transcriptional consensus signature mean *z* scores and the subgroup-differentiating signature log_2_(fold change) values. A negative correlation between both signatures indicated compound response and signature discordance, and is predictive of disruption of the expression characterizing that subgroup of tumors. Analysis was conducted and figures were generated using R and RStudio, as well as the R packages dplyr, reshape2, stringr, and pheatmap.

For sequencing analyses, HD:MB03 cells were exposed to 10 nM triptolide and total RNA was extracted with TRIzol. RNA quality was determined using an Agilent 2100 Bioanalyzer prior to performing global gene expression analysis by RNA-seq. TruSeq Stranded Total RNA-seq Library Prep kits (Illumina) were used to convert total RNA to cDNA libraries, which were then sequenced in a Genome Analyzer IIx system (Illumina). Raw reads were aligned with GRCh38/hg38 using STAR aligner (63), while raw counts were obtained using featureCounts (64) for reads mapped to each transcript. Differential expression analysis was performed using DESeq2 (65) with a covariate to adjust for batch difference. Test significance was corrected for multiple hypothesis testing and differentially expressed genes were selected with a false discovery rate of less than 0.05.

GSE85217 transcriptomic data (Cavalli et al., 2017 data set) (4) were downloaded from the GlioVis data portal (66).

The expression of genes identified in the RNA-seq analysis corresponding to each hallmark gene set was averaged for each patient in the Cavalli et al. 2017 data set (4). Patients were stratified into quartiles (Qs) based on aggregated expression, and survival was assessed using the Survminer package in R by comparing patient survival between the highest (Q4) (highest expression) and lowest quartiles (Q1) (lowest expression). The relationship between the expression of *MYC* and patient survival was compared in G3 MB samples and the other 3 major MB subgroups using the same transcriptomic data set (4). For these analyses, patients were stratified based on *MYC* expression in Qs, and patient survival in Q4 and Q1 was then compared. Cancer cell dependency scores were obtained from the combined RNAi (Broad, Novartis, Marcotte) data set included in the DepMap portal (20). A negative score indicates the gene is required for the survival of a given cancer cell line, with a lower score meaning a higher dependency. For ex vivo synergy studies, the expected drug combination responses were calculated based on a highest single agent (HSA) reference model using SynergyFinder (67). Scores under –10 denote antagonism, while values over 10 suggest a synergistic interaction.

### Statistics.

Unless otherwise indicated in the legend, results from ex vivo experiments represent the mean ± SEM of at least 3 independent experiments. Analyses of immunofluorescent staining of MB cultures were performed by quantifying positive cells per field in at least 3 high-magnification fields per condition from 3 independent experiments. For tumor size studies, tumor areas found per animal in ×2.5-magnified H&E-stained brain slides were summed and their mean ± SEM in at least 4 mice per experimental condition calculated. The size of metastatic lesions was determined by similarly measuring the area of the tumors found outside of the posterior fossa. A similar method was used to determine the size of distant metastasis found in spinal cord tissues. The number of positive cells per field in brain tumors and metastatic lesions was determined for tissue IHC quantification, and the mean ± SEM of positive cells found in at least 4 random high-magnification fields from tumors found in at least 3 different mice was determined. The significance of the 2-group analyses was determined using an unpaired, 1-tailed Student’s *t* test. Differences in multiple comparison analyses were determined using a 1-way ANOVA, followed by either a Dunnett’s or a Newman-Keuls post hoc test. Kaplan-Meier analysis was performed to assess symptom-free survival and significance was evaluated using log-rank (Mantel-Cox) tests. Unless otherwise indicated in the legend, all ex vivo data were normalized to DMSO, to a pcDNA empty vector, or to an *siRNA* scramble control, while in vivo data were normalized to 1 vehicle control. A *P* value of less than 0.05 was considered significant: *P < 0.05, **P < 0.01, ***P < 0.001, ****P < 0.0001.

### Study approval.

Mouse work was conducted in accordance with protocols approved by the Institutional Animal Care and Use Committee (IACUC) at the Medical University of South Carolina and the University of Miami.

### Data availability.

All data needed to generate the conclusions in this manuscript are present in it. Further details regarding this paper can be accessed in the supplemental materials. Additional data can be found in the supplemental Supporting Data Values file and can also be obtained from the corresponding author upon request. Analyses of triptolide response signature are available at the GitHub repository: https://github.com/RobertKSuter/Triptolide_L1000_Mb_Analysis_JCI The sequencing data of HD:MB03 cultures treated with triptolide are available at the NIH Sequence Read Archive under project ID PRJNA1079676. GSE37418 (Robinson et al., 2012) (60) and GSE85217 (Cavalli el al., 2017) (4) data sets were used in this manuscript.

## Author contributions

JRB, DJR, NGA, HJM, TB, ADC, VM, and MFR conceptualized the project. JRB, ADS, MSS, IPC, VK, PS, MTC, KP, MEV, AJH, CCC, BTH, FY, FZ, ED, AJ, and DTW contributed with methodology, investigation, and visualization. BTH analyzed cerebellar tissues from tumor-bearing mice treated with Minnelide. Bioinformatic analyses were completed by JRB, RKS, DTW, YB, FY, NGA, and XSC, while animal studies were performed by JRB, ADS, MSS, and FZ. The manuscript was written by JRB, DJR, MFR, CCC, HJM, and RV, and supervised by corresponding author JRB.

## Supplementary Material

Supplemental data

Unedited blot and gel images

Supporting data values

## Version 1 06/17/2024

In-Press Preview

## Version 2 08/01/2024

Electronic publication
