# Supplementary material for: Triptolide and its prodrug Minnelide target high-risk MYC-amplified medulloblastoma in preclinical models
Source: J Clin Invest. 2024 Jun 17;134(15):e171136. doi: 10.1172/JCI171136 (PMC11290968; doi:10.1172/JCI171136)
Supplement: Supplemental data [file jci-134-171136-s009.pdf]

Supplemental Table 1

Antibody	Supplier	Catalog number	Usage
Anti-Mouse IgG	Cell Signaling Technology	7076	Immunoblotting
Anti-Rabbit IgG	Cell Signaling Technology	7074	Immunoblotting
Anti-Mouse IgG	Cell Signaling Technology	8125	IHC
Anti-Rabbit IgG	Cell Signaling Technology	8114	IHC
Cleaved Casp3	Cell Signaling Technology	9661	IHC & Immunoblotting
GAPDH	Cell Signaling Technology	51332	Immunoblotting
β-Tubulin	Cell Signaling Technology	2144	Immunoblotting
β-Actin	Cell Signaling Technology	4970	Immunoblotting
Ki67	Abcam	ab15580	IHC
MYC	Cell Signaling Technology	18583	Immunoblotting
MYC	Abcam	ab32072	Immunoprecipitation & IHC
Phospho MYC (Ser62)	Cell Signaling Technology	13748	Immunoblotting
Phospho MYC (Thr58)	Cell Signaling Technology	46650	Immunoblotting
Ubiquitin	Cell Signaling Technology	3936	Immunoblotting
RPB1	Cell Signaling Technology	2629	Immunoblotting
Phospho RPB1 (Ser2/Ser5)	Cell Signaling Technology	13546	Immunoblotting
HA-Tag	Cell Signaling Technology	3724	Immunoblotting

Supplemental Table 1: List of Antibodies utilized in this manuscript.

Supplemental Figure 1

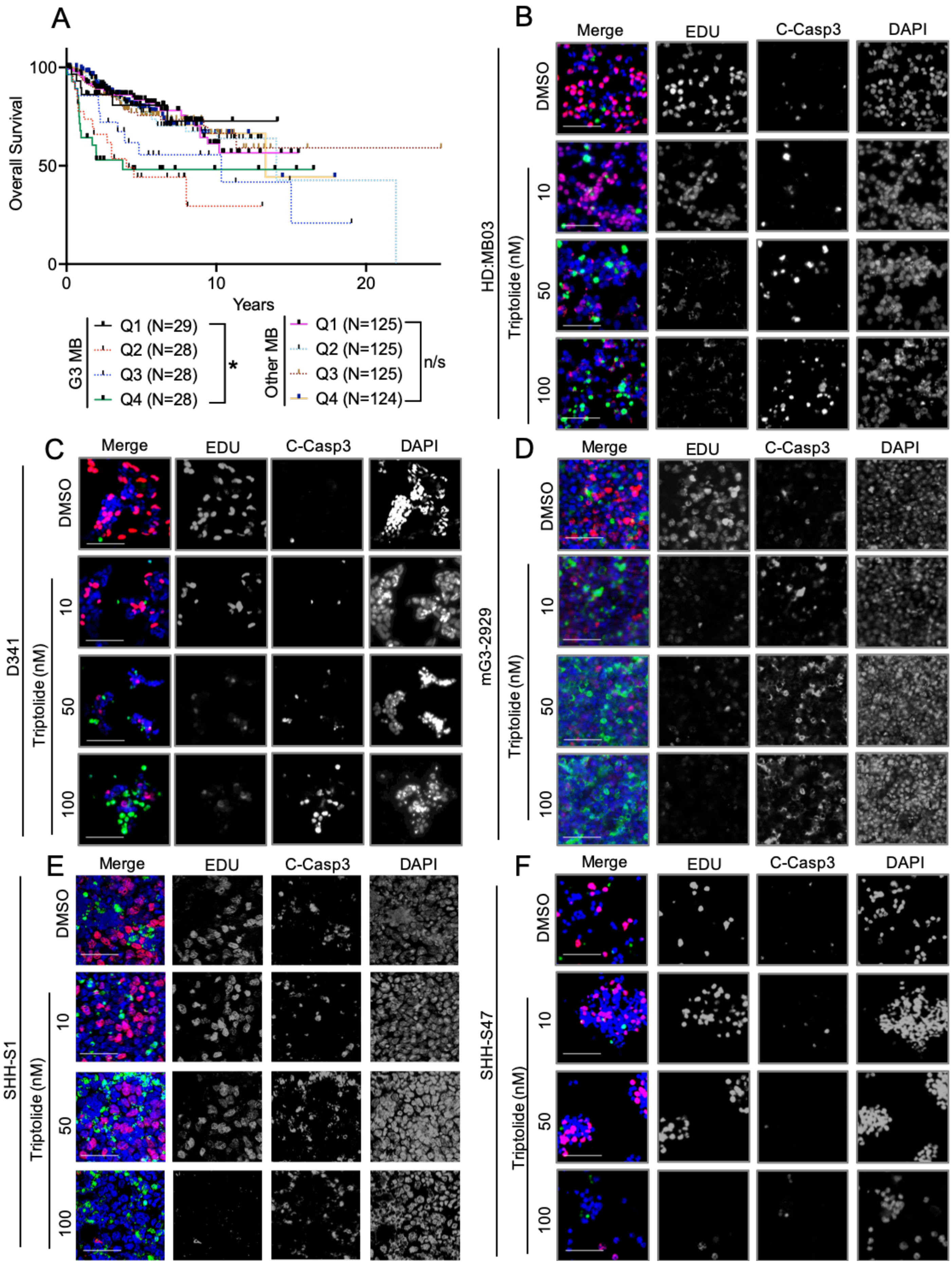

Supplemental Figure 1: (**A**) Cavalli et al., 2017 transcriptomic data was used to compare the correlation between *MYC* expression and survival in G3 MB, as well as in the rest of the MB subgroups. Patients were stratified in quartiles (Q) based on *MYC* expression. Statistical significance between low (Q1) and high (Q4) *MYC* expressing patients was assessed using Log-rank (Mantel-Cox) tests. (**B**) HD:MB03 cultures were exposed for 16 hours to indicated concentrations of triptolide. Cell proliferation was assayed by EdU incorporation, while apoptosis levels were determined by C-Casp3 staining. (**C**) D341 cultures were similarly exposed to triptolide and cell proliferation and death determined. (**D**) Proliferation and cell death were similarly determined in triptolide-treated mG3-2929 cultures. **(E**) SHH-S1 cultures were exposed to triptolide for 16 hours prior to similarly determine cell proliferation and cell death. (**F**) Proliferation and cell death were determined in similarly treated SHH-S47 cultures. Representative images (scale bar: 50 µm) of the single and merged channels of images in Figure 2D are shown. * $p< 0.05$, ** $p< 0.01$, *** $p< 0.001$, **** $p< 0.0001$, n/s non-significant.

Supplemental Figure 2

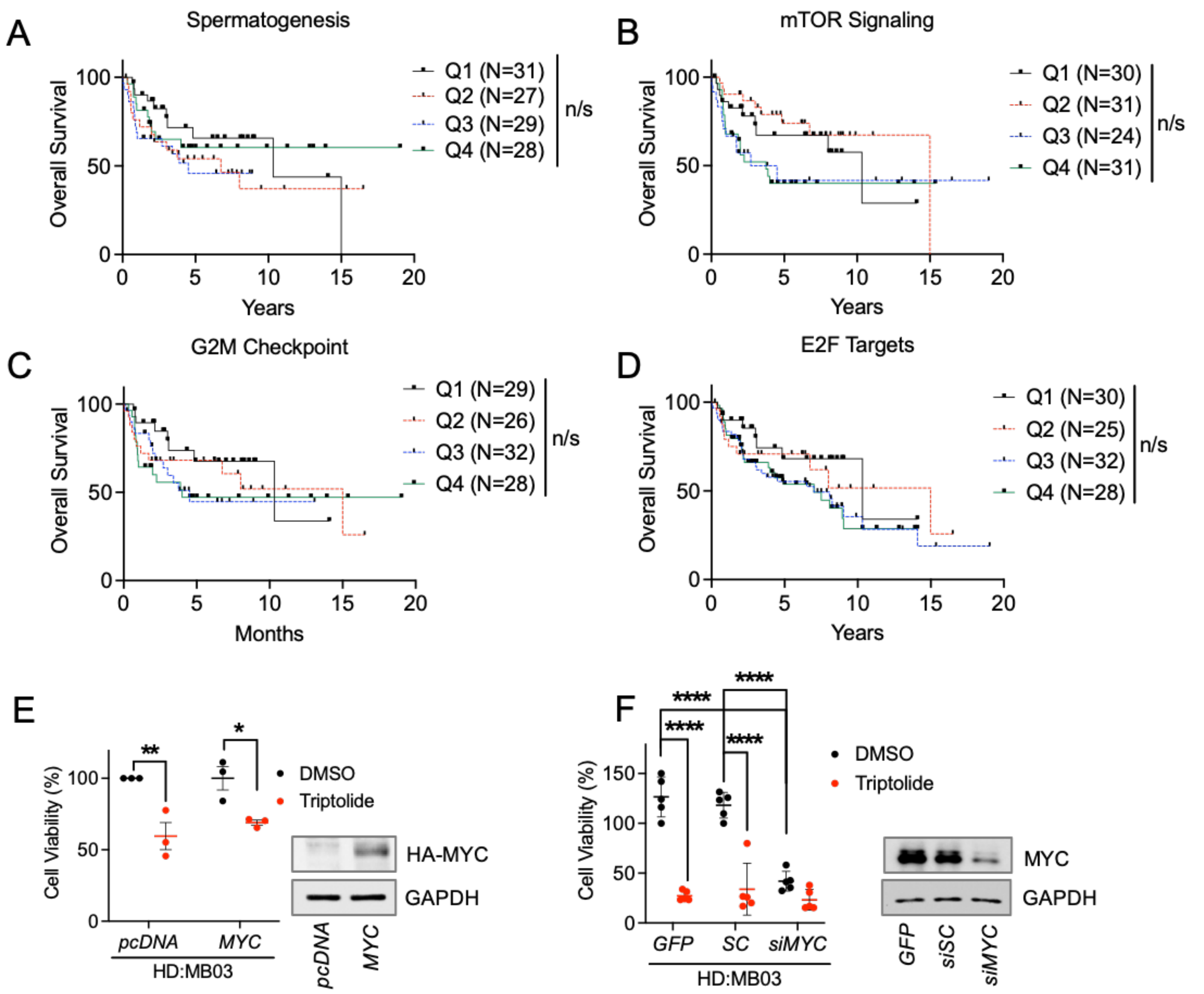

Supplemental Figure 2: (**A**) The correlation between the expression of the spermatogenesis hallmark and patient survival in G3 MB patients was determined using transcriptomic data from the Cavalli et al., 2017 dataset. The average expression of each gene within the hallmark gene set was calculated for each patient. Patients were then stratified into quartiles (Q) based on this averaged gene set expression, and overall survival was analyzed using Log-rank (Mantel-Cox) tests. (**B**) Similar correlation studies in G3 MB patients were performed for mTOR signaling. Overall survival was analyzed using Log-rank (Mantel-Cox) tests. (**C**) The correlation between the expression of the G2/M checkpoint hallmark and patient survival in G3 MB patients was similarly

determined. Overall survival was analyzed using Log-rank (Mantel-Cox) tests. (**D**) The correlation between expression and G3 MB patient survival was similarly determined for the E2F targets hallmark. Overall survival was analyzed using Log-rank (Mantel-Cox) tests. (**E**) HD:MB03 cultures were transfected with a *HA-MYC* construct 72h before treatment with 50 nM triptolide. Cell viability was assessed by MTT reduction 48h later (n=3). MYC levels were determined by immunoblotting 72h after transfection. Mean ± SEM of data normalized to DMSO was analyzed using an unpaired Student's t-test. (**F**) HD:MB03 cells were similarly transfected for 48h before being treated with 50 nM triptolide. Cell viability was assessed by MTT reduction 48h later. A representative experiment of n=3 in which mean ± SD was analyzed using a one-way ANOVA followed by Newman-Keuls post-hoc test is shown. MYC levels were evaluated by immunoblotting 72h after transfection. In all cases, images of representative immunoblottings are shown. * p< 0.05, ** p< 0.01, *** p< 0.001, **** p< 0.0001, n/s non-significant.

Supplemental Figure 3

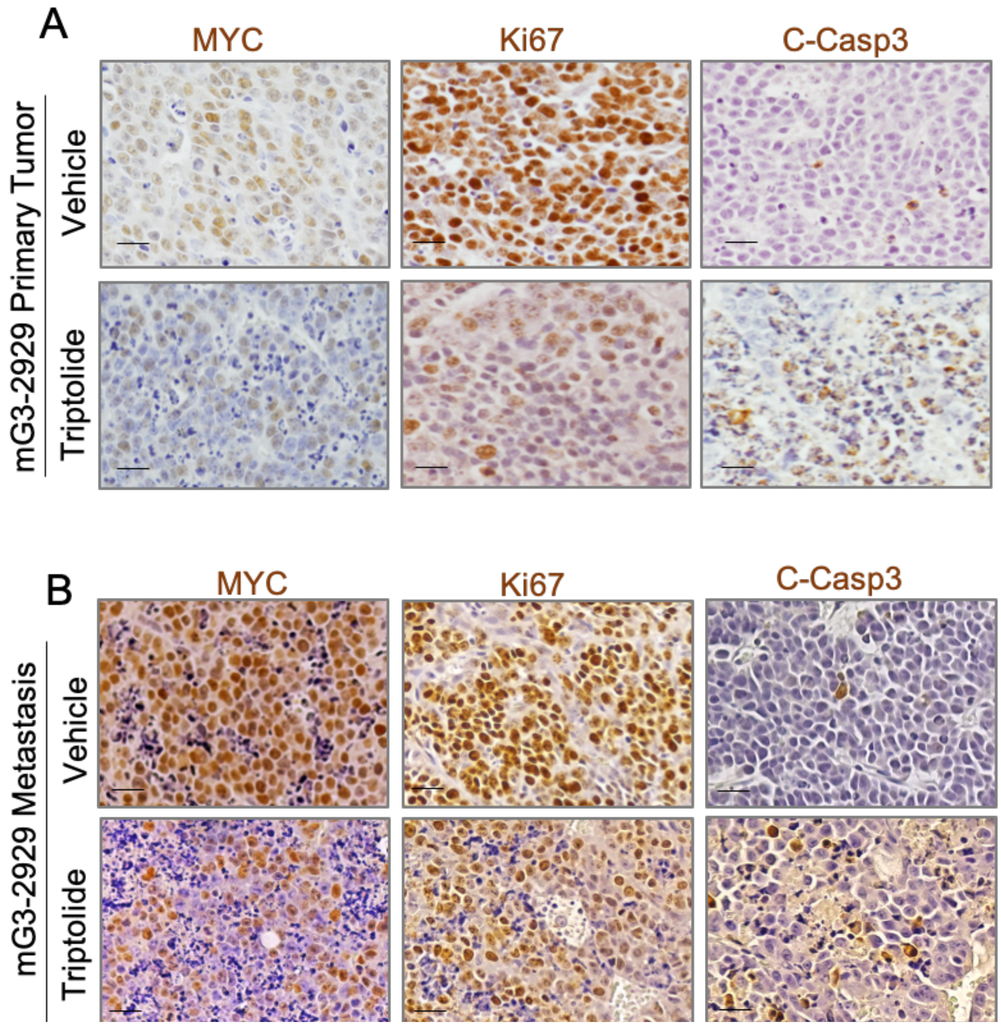

Supplemental Figure 3: (**A**) Mice harboring mG3-2929 tumors were treated with triptolide (0.4 mg/kg, i.p., daily) for 7 days prior to harvesting brain tissues and staining them using antibodies against indicated proteins. A larger field for the representative images of tumors in the posterior fossa included in Figure 5D is shown. (**B**) Mice harboring mG3-2929 tumors were similarly treated. A larger field for the representative images of tumors found outside of the posterior fossa included in Figure 6F is shown.

Supplemental Figure 4

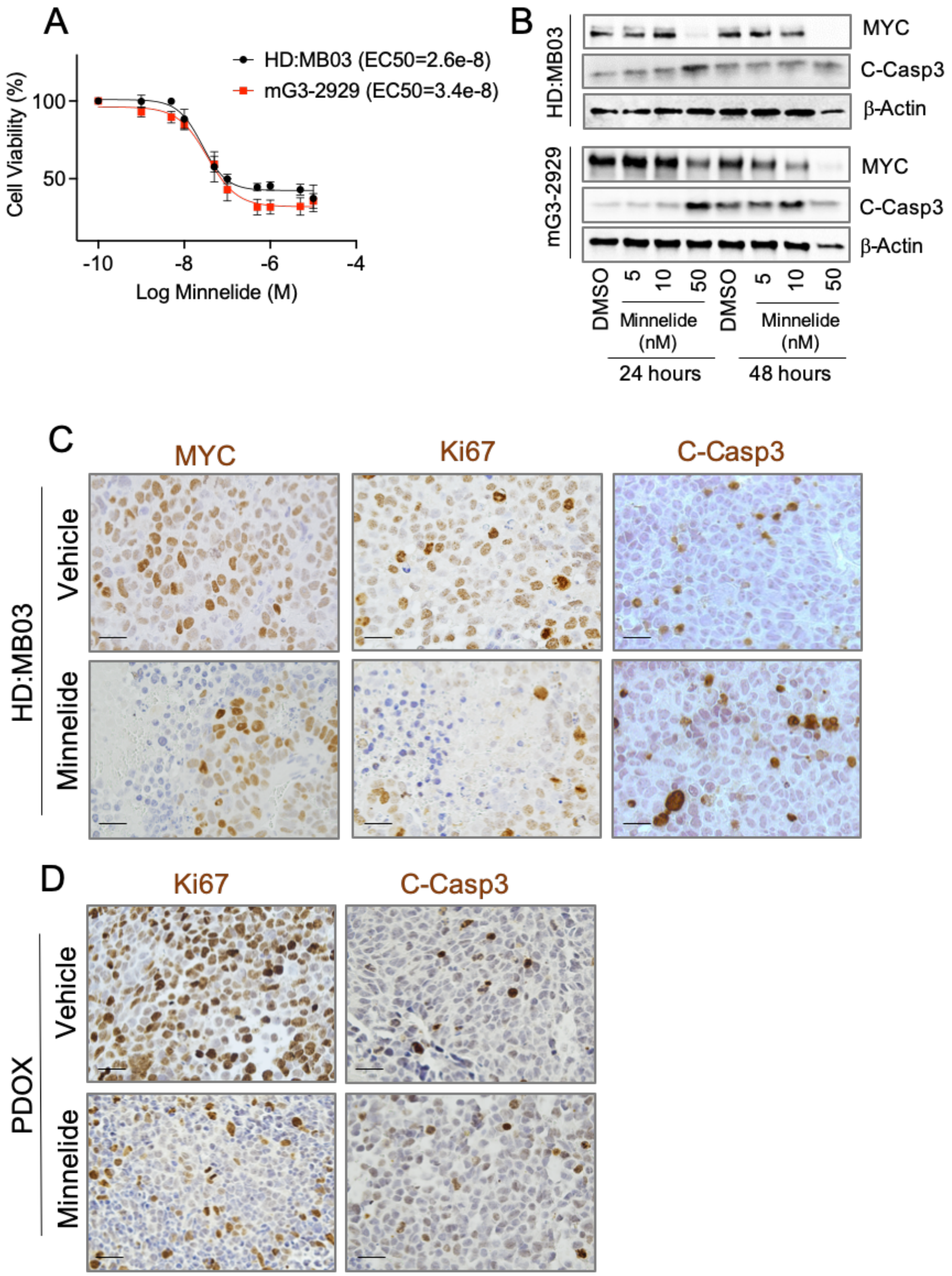

Supplemental Figure 4: (**A**) G3 MB cultures were exposed to Minnelide in the presence of antarctic phosphatase (2 units/mL) for 48h before assessing cell viability by MTT reduction. Non-linear regression analyses were performed and the mean ± SEM of data normalized to DMSO is shown (HD:MB03 n=3, mG3-2929 n=4). (**B**) Similar cultures were exposed to Minnelide and the levels of the indicated proteins in their lysates

determined by immunoblotting. Images of representative immunoblottings are shown. (**C**) Mice harboring HD:MB03 tumors were treated with Minnelide (0.4 mg/kg, i.p., daily) for 7 days prior to harvesting brain tissues and staining them using antibodies against indicated proteins. A larger field for the representative images of tumors included in Figure 7E is shown. (**D**) Mice harboring RCMB28 tumors were similarly treated. A larger field for the representative images of tumors found outside of the posterior fossa included in Figure 8B is shown.

Supplemental Figure 5

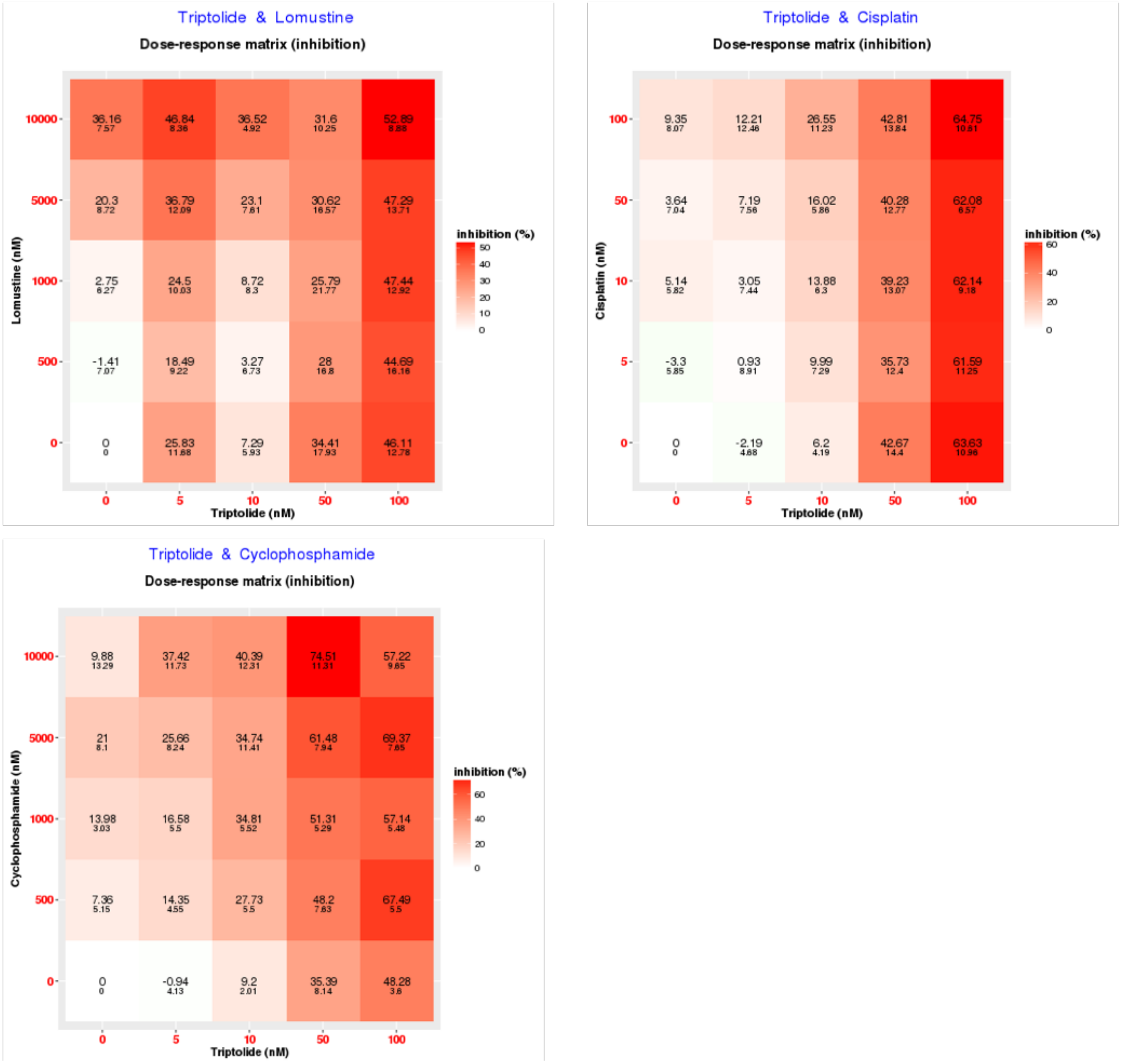

Supplemental Figure 5: mG3-2929 cultures were exposed to increasing concentrations of triptolide alone or in combination with either lomustine, cisplatin or cyclophosphamide. Cell viability was determined by an MTT reduction assay, and data were analyzed using SynergyFinder to assess the synergistic scores of these drug combinations. Heatmaps showing percentage of growth inhibition for the combination of each drug concentration are shown (lomustine/cisplatin n=3, cyclophosphamide n=4).

Supplemental Figure 6

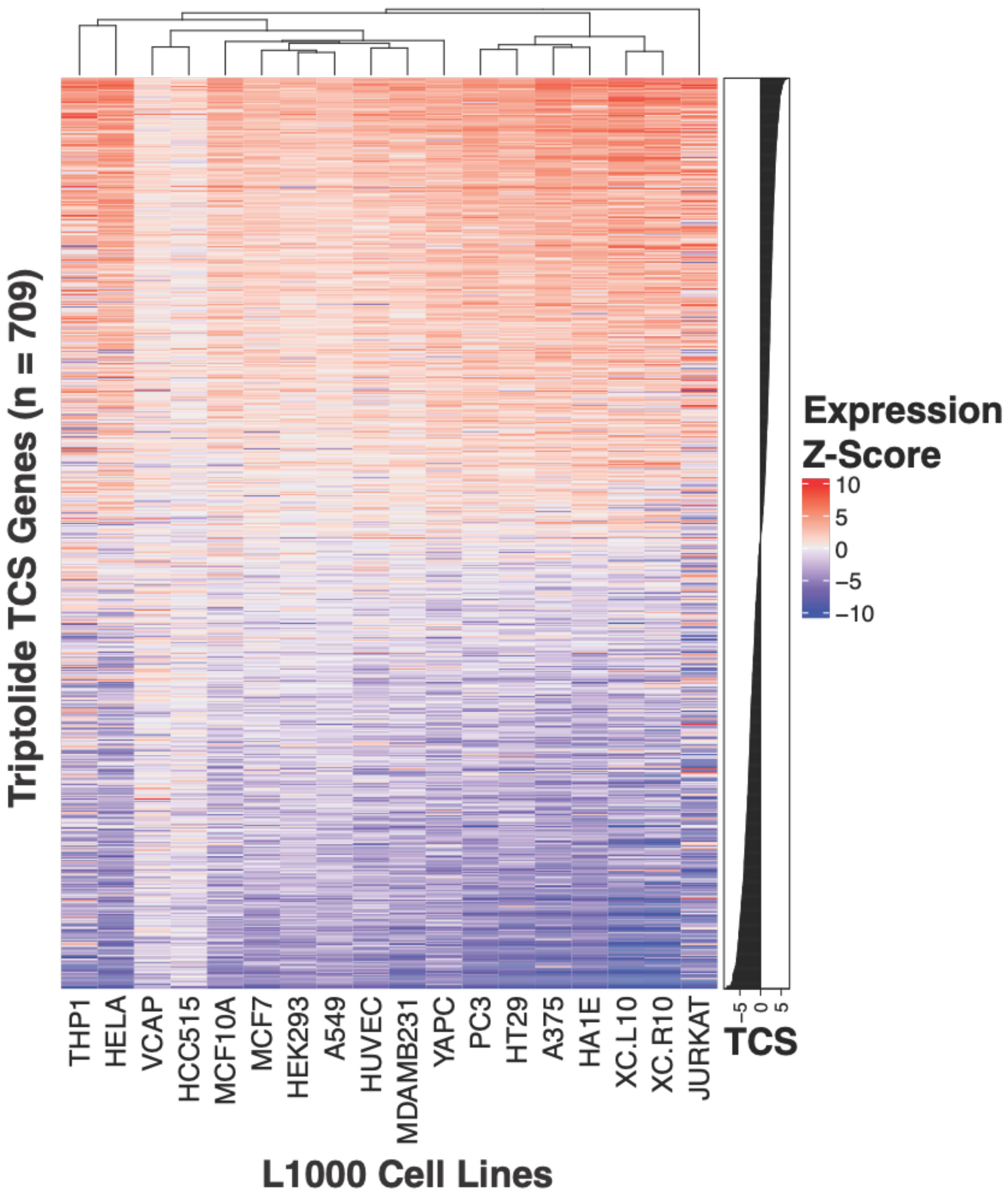

Supplemental Figure 6: Heatmap showing the overall transcriptional consensus signature (TCS) for triptolide derived from the LINCS L1000 dataset. Row annotation bar plot depicts the final magnitude and direction (row sums) for each gene within the triptolide TCS. Level 5 processed data (Z-scores) from LINCS dataset LDS-1613 were downloaded for processing. The data were subsetted for 24h treatment times, and gene expression profiles were aggregated for samples within the same cell line (technical/biological replicates and/or different doses of triptolide). Genes with a Z-Score > 1 in more than 30% of the triptolide-treated cell lines were included in the triptolide signature. The resulting TCS depicted in Figure 1A is the intersection of this triptolide TCS. Genes

differentially expressed between each MB subgroup relative to the rest of the MB subgroups within the dataset are shown in Figure 1C.
