# Supplementary material for: Triptolide and its prodrug Minnelide target high-risk MYC-amplified medulloblastoma in preclinical models
Source: J Clin Invest. 2024 Jun 17;134(15):e171136. doi: 10.1172/JCI171136 (PMC11290968; doi:10.1172/JCI171136)

Figure 2

Full unedited blot for Figure 2B
G3 MB vs. SHH MB – MYC (57-65 kDa)

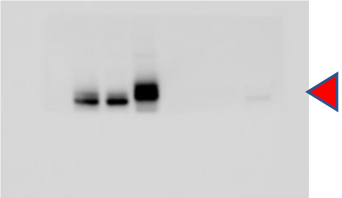

Full unedited blot for Figure 2B
G3 MB vs. SHH MB – GAPDH (37 kDa)

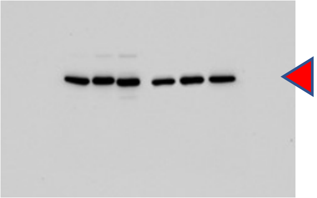

Figure 3

Full unedited blot for Figure 3E
HD:MB03
MYC (57-65 kDa), Cleaved casp-3 (17, 19 kDa)

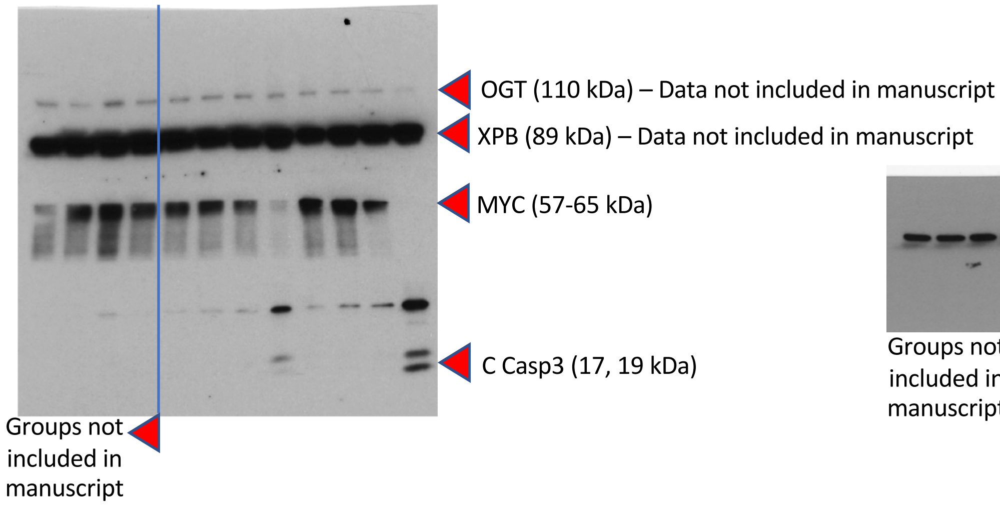

Full unedited blot for Figure 3E
HD:MB03
GAPDH (37 kDa)

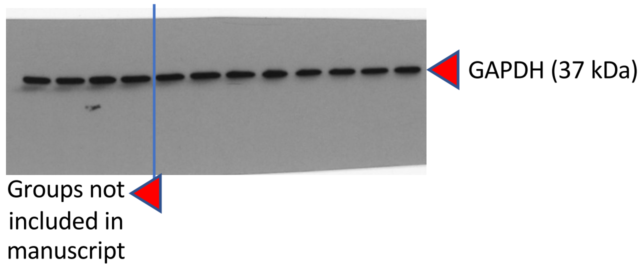

Full unedited blot for Figure 3E
D341
MYC (57-65 kDa)

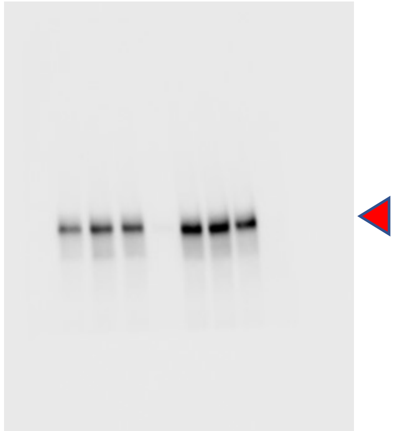

Full unedited blot for Figure 3E
D341
Cleaved casp-3 (17, 19 kDa)

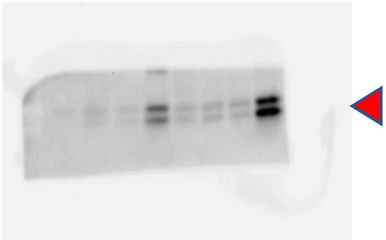

Full unedited blot for Figure 3E
D341
GAPDH (37 kDa)

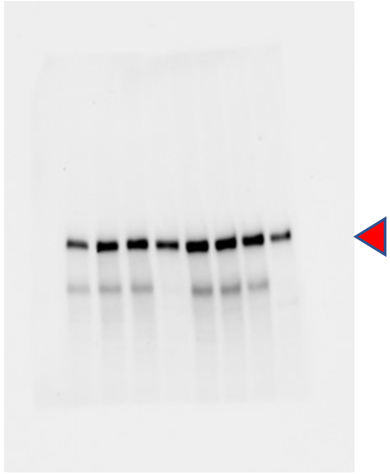

Full unedited blot for Figure 3E
mG3-2929
MYC (57-65 kDa)

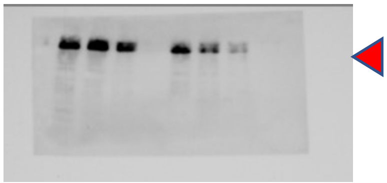

Full unedited blot for Figure 3E
mG3-2929
Cleaved casp-3 (17, 19 kDa)

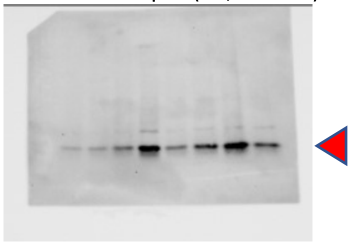

Full unedited blot for Figure 3E
mG3-2929
GAPDH (37 kDa)

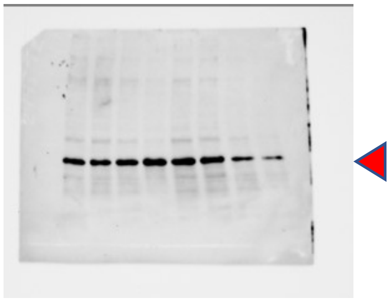

Figure 3 and Supplemental Figure 2

Full unedited blot for Figure 3F

mG3-2929
MYC (57-65 kDa)

mG3-2929
GAPDH (37 kDa)

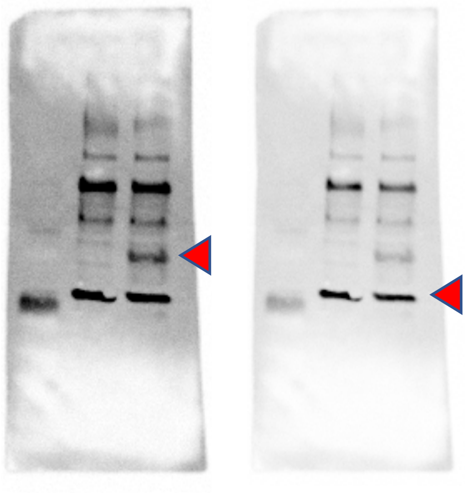

Full unedited blot for Supplemental Figure 2E

HD:MB03
MYC (57-65 kDa)

HD:MB03
GAPDH (37 kDa)

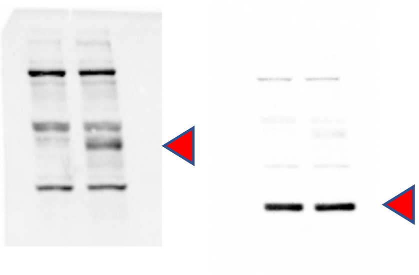

Full unedited blot for Figure 3G
mG3-2929
MYC (57-65 kDa)

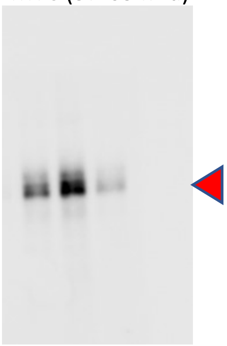

Full unedited blot for Figure 3G
mG3-2929
GAPDH (37 kDa)

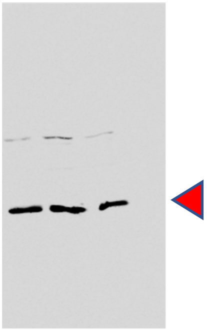

Full unedited blot for Supplemental Figure 2F

HD:MB03
MYC (57-65 kDa)

HD:MB03
GAPDH (37 kDa)

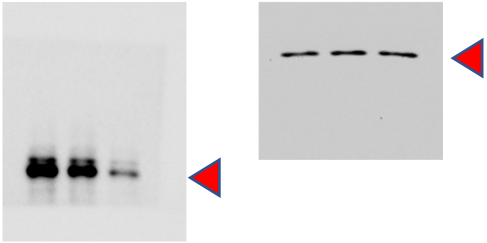

Full unedited blot for Figure 3H
SHH-S47
MYC (57-65 kDa)

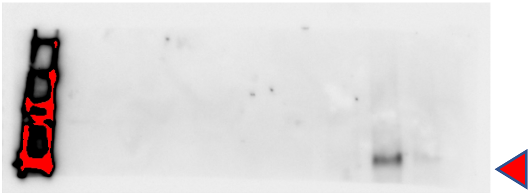

Full unedited blot for Figure 3H
SHH-S47
GAPDH (37 kDa)

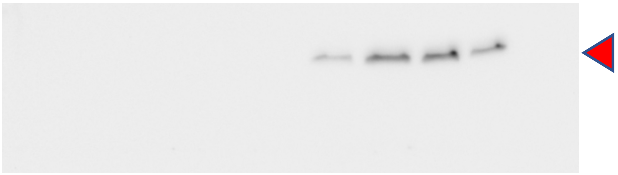

Figure 4

Full unedited blot for Figure 4B
HD:MB03
MYC (57-65 kDa)

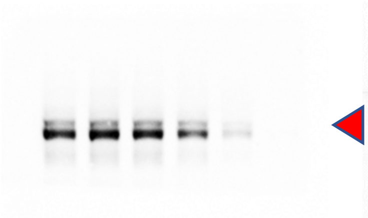

Full unedited blot for Figure 4B
HD:MB03
P-RPB1 (250 kDa)

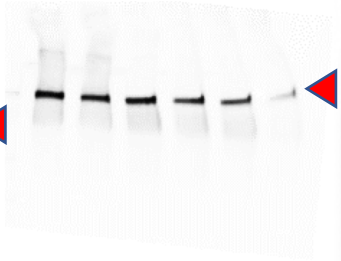

Full unedited blot for Figure 4B
HD:MB03
RPB1 (250 kDa)

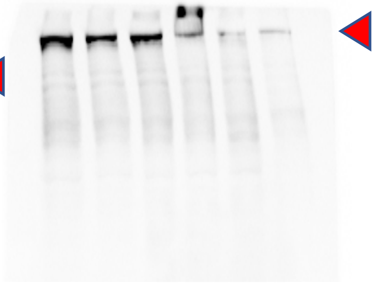

Full unedited blot for Figure 4B
HD:MB03
GAPDH (37 kDa)

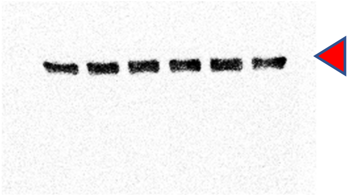

Full unedited blot for Figure 4E
HD:MB03
RPB1 (250 kDa)

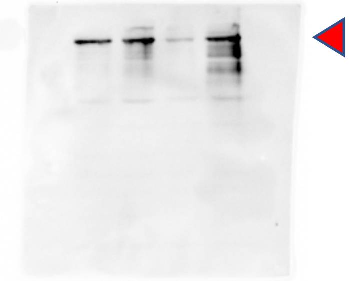

Full unedited blot for Figure 4E
HD:MB03
β-Tubulin (55 kDa)

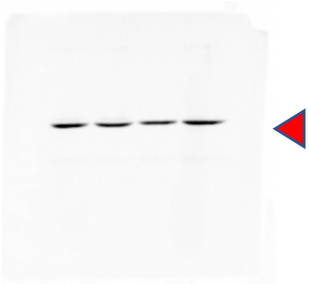

Full unedited blot for Figure 4H
HD:MB03
MYC (57-65 kDa)

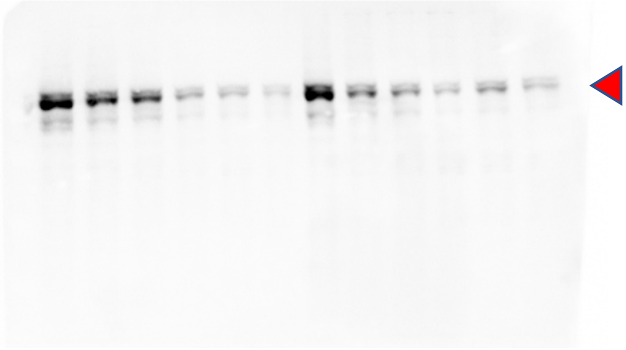

Full unedited blot for Figure 4H
HD:MB03
GAPDH (37 kDa)

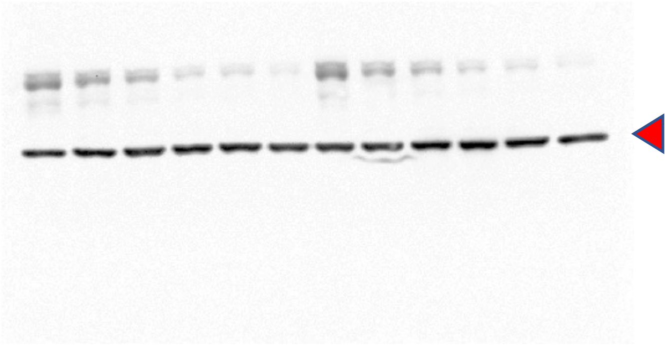

Figure 4

Full unedited blot for Figure 4I
HD:MB03
P-MYC (Ser62) (62 kDa)

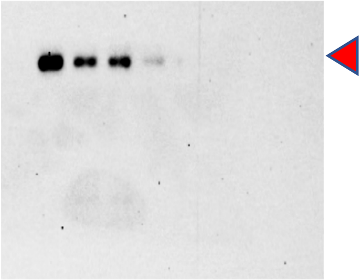

Full unedited blot for Figure 4I
HD:MB03
P-MYC (Thr58) (62 kDa)

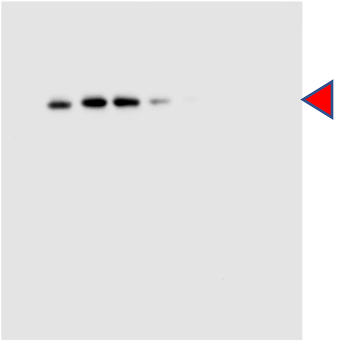

Full unedited blot for Figure 4I
HD:MB03
MYC (57-65 kDa)

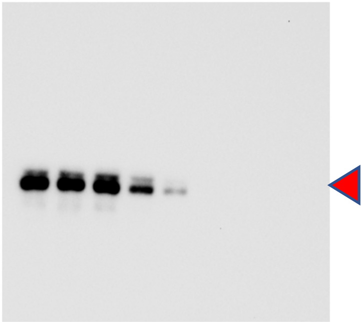

Full unedited blot for Figure 4I
HD:MB03
GAPDH (37 kDa)

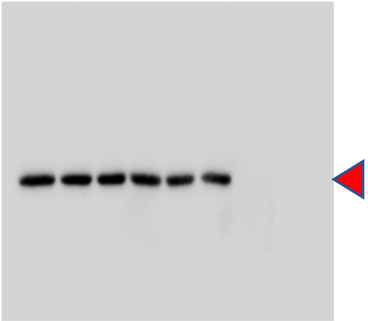

Figure 4

Full unedited blot for Figure 4J
HD:MB03
Ubiquitin

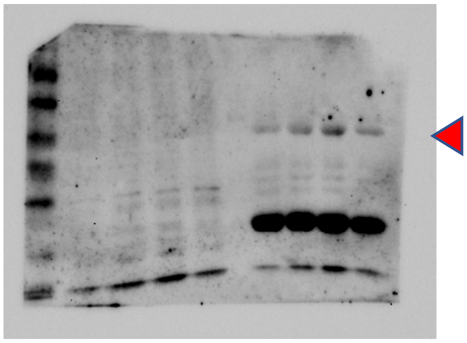

Full unedited blot for Figure 4J
HD:MB03
GAPDH (37 kDa)

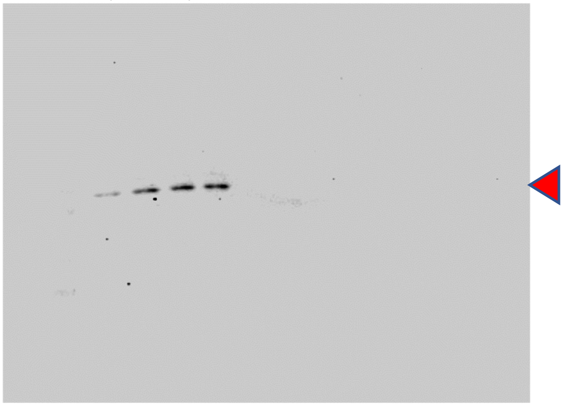

Full unedited blot for Figure 4J
HD:MB03
MYC (57-65 kDa)

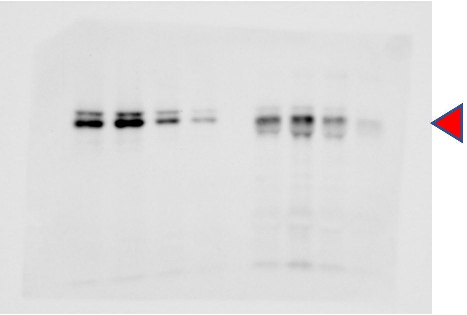

Full unedited blot for Figure 4K
HD:MB03
MYC (57-65 kDa)

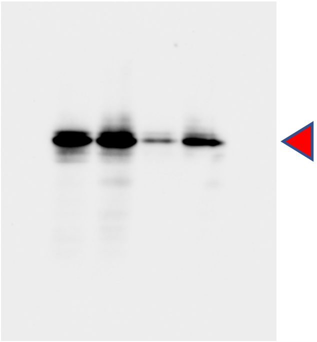

Full unedited blot for Figure 4K
HD:MB03
GAPDH (37 kDa)

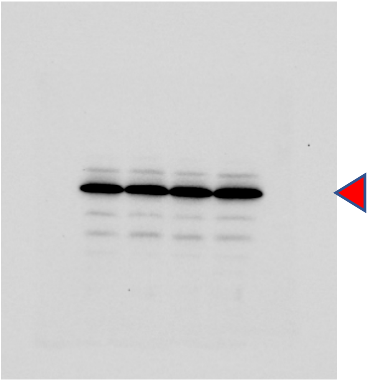

Figure 5 and Figure 6

Full unedited gel for Figure 5C
mG3-2929 tumor
MYC (57-65 kDa)

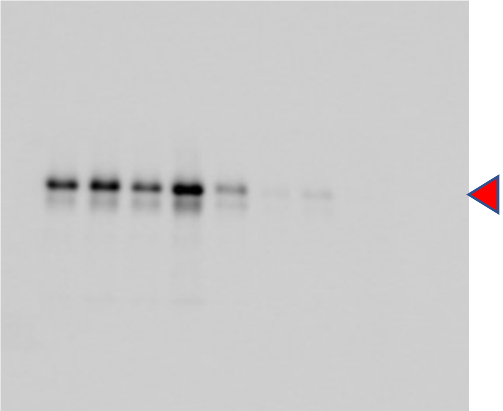

Full unedited gel for Figure 5C
mG3-2929 tumor
GAPDH (37 kDa)

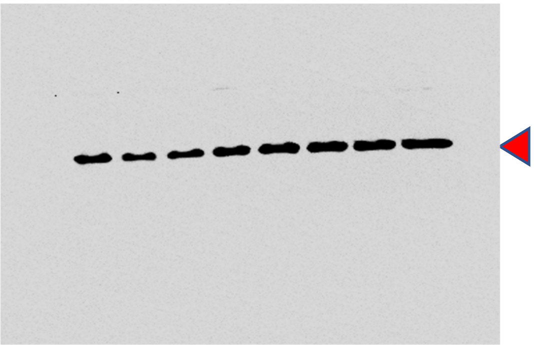

Full unedited blot for Figure 6A
D425
MYC (57-65 kDa)

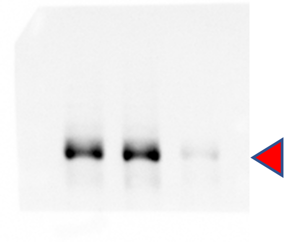

Full unedited blot for Figure 6A
D458
MYC (57-65 kDa)

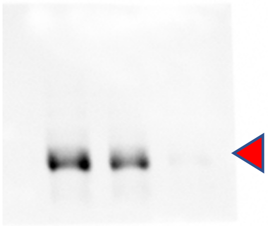

Full unedited blot for Figure 6A
D425
GAPDH (37 kDa)

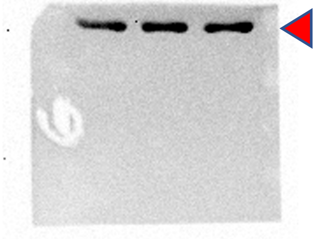

Full unedited blot for Figure 6A
D458
GAPDH (37 kDa)

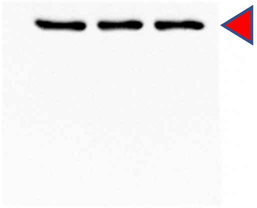

Supplemental Figure 4

Full unedited gel for Supplemental Figure 4B
HD:MB03
MYC (57-65 kDa)

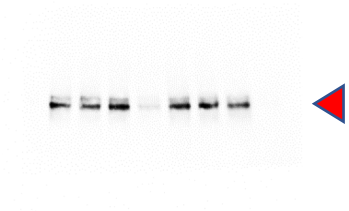

Full unedited gel for Supplemental Figure 4B
HD:MB03
Cleaved casp-3 (17, 19 kDa)

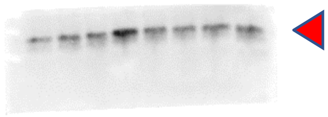

Full unedited gel for Supplemental Figure 4B
HD:MB03
β-actin (45 kDa)

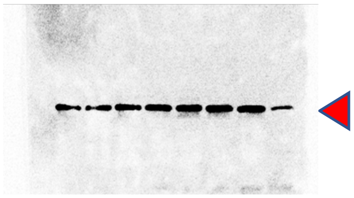

Full unedited blot for Supplemental Figure 4B
mG3-2929
MYC (57-65 kDa)

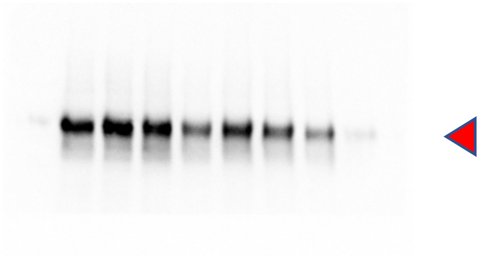

Full unedited blot for Supplemental Figure 4B
mG3-2929
Cleaved casp-3 (17, 19 kDa)

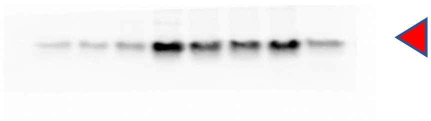

Full unedited blot for Supplemental Figure 4B
mG3-2929
β-actin (45 kDa)

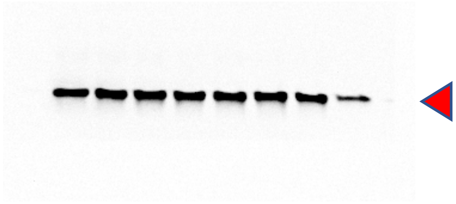

Supplement: Unedited blot and gel images [file jci-134-171136-s010.pdf]
